# The acyl-CoA-binding protein VdAcb1 is essential for carbon starvation response and contributes to virulence in *Verticillium dahliae*

**DOI:** 10.1007/s42994-024-00175-3

**Published:** 2024-07-13

**Authors:** Jing Zhuang, Ya-Duo Zhang, Wei-Xia Sun, Juan Zong, Jun-Jiao Li, Xiao-Feng Dai, Steven J. Klosterman, Jie-Yin Chen, Li Tian, Krishna V. Subbarao, Dan-Dan Zhang

**Affiliations:** 1 State Key Laboratory for Biology of Plant Diseases and Insect Pests, Institute of Plant Protection, Chinese Academy of Agricultural Sciences, Beijing, 100193 China; 2 School of Life Science, Qufu Normal University, Qufu, 273165 China; 3 Shenzhen Branch, Guangdong Laboratory of Lingnan Modern Agriculture, Key Laboratory of Synthetic Biology, Ministry of Agriculture and Rural Affairs, Agricultural Genomics Institute at Shenzhen, Chinese Academy of Agricultural Sciences, Shenzhen, 518120 China; 4 Western Agricultural Research Center, Chinese Academy of Agricultural Sciences, Changji, 831100 China; 5 United States Department of Agriculture, Agricultural Research Service, Salinas, CA 93905 USA; 6 Department of Plant Pathology, University of California, Davis, c/o United States Agricultural Research Station, Salinas, CA 93905 USA

**Keywords:** *Verticillium dahliae*, Unconventional secreted protein, Acyl-CoA-binding protein, Carbon starvation, Virulence factor

## Abstract

In the face of carbon, nitrogen, and phosphorus starvation, microorganisms have evolved adaptive mechanisms to maintain growth. In a previous study, we identified a protein predicted to contain acyl-CoA-binding domains in the plant pathogenic fungus *Verticillium dahliae*. The predicted protein, designated VdAcb1, possesses an atypical signal peptide. However, the functions of this acyl-CoA-binding protein in *V. dahliae* are not clear. In this research, in vivo or in vitro assays confirmed that VdAcb1 is secreted extracellularly from *V. dahliae*, although it does not have the typical signal peptide. Furthermore, the unconventional secretion of VdAcb1 was dependent on VdGRASP, a member of the compartment for unconventional protein secretion (CUPS). The deletion mutant strain of *VdAcb1* (Δ*VdAcb1)* exhibited significant sensitivity to carbon starvation. RNA-seq revealed that the expression of genes related to filamentous growth (MSB2 pathway) and sugar transport were regulated by *VdAcb1* under conditions of carbon starvation. Yeast one-hybrid experiments further showed that the expression of VdAcb1 was positively regulated by the transcription factor VdMsn4. The Δ*VdAcb1* strain showed significantly reduced virulence on *Gossypium hirsutum* and *Nicotiana benthamiana*. We hypothesize that under conditions of carbon starvation, the expression of *VdAcb1* is activated by VdMsn4 and VdAcb1 is secreted into the extracellular space. In turn, this activates the downstream MAPK pathway to enhance filamentous growth and virulence of *V. dahliae*.

**Supplementary Information:**

The online version contains supplementary material available at 10.1007/s42994-024-00175-3.

## Introduction

Nutrient utilization is critically important for plant pathogens to infect and colonize host tissues. Successful pathogens have evolved mechanisms to manipulate the host to survive in a nutrient-poor environment. Pathogens infect plants in an environment lacking adequate nutrients, such as carbon, nitrogen, or phosphorus (Solomon et al. 2003; Thomas et al. 2001). Carbon is the most basic element to maintain biological life activities, and thus sufficient acquisition of sugars from plants is critical for successful invasion. In turn, the plant may modulate carbohydrate availability while activating defenses (Tauzin and Giardina 2014). The wheat stripe rust pathogen *Puccinia striiformis* f. sp. *tritici (Pst)* can uptake sucrose from the environment during infection by up-regulating the expression of hexose transporters and decomposing it by the unique invertase PsINV (Chang et al. 2017, 2020). Thus, nutrition is an important factor in regulating host–pathogen interactions.

Eukaryotes sense changes in the external environment and make required adjustments depending on the support by cell surface receptors and internal signal transduction networks (Carraway et al. 2003). Cell surface receptors usually couple downstream conserved signaling pathways, including the Ras/cAMP-PKA and the mitogen-activated protein kinase (MAPK) cascades, to regulate cell adaptability to stressful environments (Hamel et al. 2012; Lafon et al. 2006; Zhao et al. 2005). Signaling mucins are macromolecular transmembrane glycoproteins, which can sense external nutritional dynamics in the environment, and transmit signals to the interior of the cell (Carraway et al. 2003). In *Aspergillus nidulans*, starvation leads to the constitutive activation of the signaling mucin MsbA, which enhances cellulase activity by increasing the secretion of the cellobiohydrolase CbhA and improving substrate attachment, enhancing the starvation response (Brown et al. 2014). In *Saccharomyces cerevisiae*, nutrient deprivation activates the MAPK cascade modulating filamentous and invasive growth, and biofilm formation (Cullen et al. 2000; Karunanithi and Cullen 2012). And the starvation signal can be transmitted to transcription factors, through the cAMP-PKA signaling pathway, to regulate the expression of some genes related to nutrient utilization (Rolland et al. 2002). For example, when glucose is deficient, the transcription factor MSN2 is phosphorylated by PKA and then transferred from the cytoplasm to the nucleus, where it then regulates the expression of some metabolism-related genes in response to starvation (Mayordomo et al. 2002).

*Verticillium dahliae* is a notorious soil-borne phytopathogenic fungus that invades the roots and colonizes the xylem (Klosterman et al. 2009; Zhang et al. 2022). Verticillium wilt, caused by *V. dahliae*, is a major disease on cotton and other high value crops (Fradin and Thomma 2006). The mechanisms by which *V. dahliae* adapts to carbon starvation remain poorly characterized, although some starvation stress-responsive genes have been reported in *V. dahliae*. For example, VdNUC-2 is involved in phosphate-responsive signaling in *V. dahliae* and is required for the full virulence (Deng et al. 2015). The bZip transcription factor VdHapX is a conserved protein that mediates adaptation to iron starvation, affects microsclerotium formation, and is crucial for virulence of *V. dahliae* (Wang et al. 2018b). Detection of differentially expressed genes induced by carbon starvation, genes involved in utilization or production of acetyl-CoA, glycolysis, fatty acid biosynthesis or metabolism, and carbohydrate degradation can all be induced (Coradetti et al. 2013; Glass et al. 2013).

The acyl-CoA-binding protein (ACBP) contains a highly conserved ACBP domain, which plays an important role in acyl-CoA binding and transport (Fan et al. 2010; Xiao and Chye 2011). The basic function of the ACBP containing protein (ACB1) is to bind acyl-CoA and regulate other metabolic pathways (Schjerling et al. 1996). The acylation process is necessary for fatty acid production and oxidation. Long-chain acyl-CoA is a by-product of lipid metabolism and can also function as a signal molecule (Roduit et al. 2004). Whether in eukaryotes or prokaryotes, acetyl-CoA binding protein participation in lipid metabolism has been widely reported (Burton et al. 2005; Ferreira et al. 2017; Nie et al. 2018). Mutation of the *Acb1* gene in yeast results in stearoyl-CoA accumulation in cells, and myristoyl-CoA, palmitoyl-CoA, and oleoyl-CoA were significantly reduced (Knudsen et al. 1994; Mandrup et al. 1993). MrACBP from the fungus *Monascus ruber* preferentially binds to myristoyl-CoA and can affect *Monascus* pigment biosynthesis (Long et al. 2018). In *Magnaporthe oryzae,* disruption of MoAcb1 causes delayed hyphal growth, significant reduction in conidial production and glycogen availability, delayed appressorium development, and reduced pathogenicity (Cao et al. 2023). Investigation of the roles of two ACBPs, PsACBP1 and PsACBP2, revealed that both are required for asexual development and virulence in *Phytophthora sojae* (Pei et al. 2022).

The acetyl-CoA binding protein is one of the most studied unconventionally secreted proteins. Acb1 lacks the conventional signal sequence targeting the ER. However, when yeast is cultured on media containing potassium acetate, Acb1 is secreted due to lack of nutrition (Charmpilas et al. 2020). There are two kinds of ACBP family proteins in *A. oryzae*, and AoAcb2 can also be secreted into the supernatant by an unconventional secretion mechanism under carbon starvation conditions (Kwon et al. 2017). Secretion of either Acb1 in *S. cerevisiae* or AcbA in *D. discoideum* are dependent on a Golgi membrane-associated protein (GRASP) (Manjithaya et al. 2010). And the secretion of Acb1 in *S. cerevisiae* also depends on autophagy genes and the plasma membrane t-SNARE, Sso1 (Duran et al. 2010).

In our previous research, a predicted *V. dahliae* acyl-CoA-binding protein (VdAcb1), lacking a signal peptide, was detected in the exoproteome of the fungus following its induction in cotton tissue culture medium (Chen et al. 2016). However, the function of VdAcb1 is not clear in *V. dahliae*. The objectives of this study were to: (1) confirm that VdAcb1 is secreted and to characterize the mechanism of its secretion; (2) determine whether VdAcb1 is involved in the response to starvation; and (3) ascertain the role of VdAcb1 in *V. dahliae* virulence.

## Results

### *VdAcb1* encodes the acyl-CoA-binding protein

**Fig. 1:**
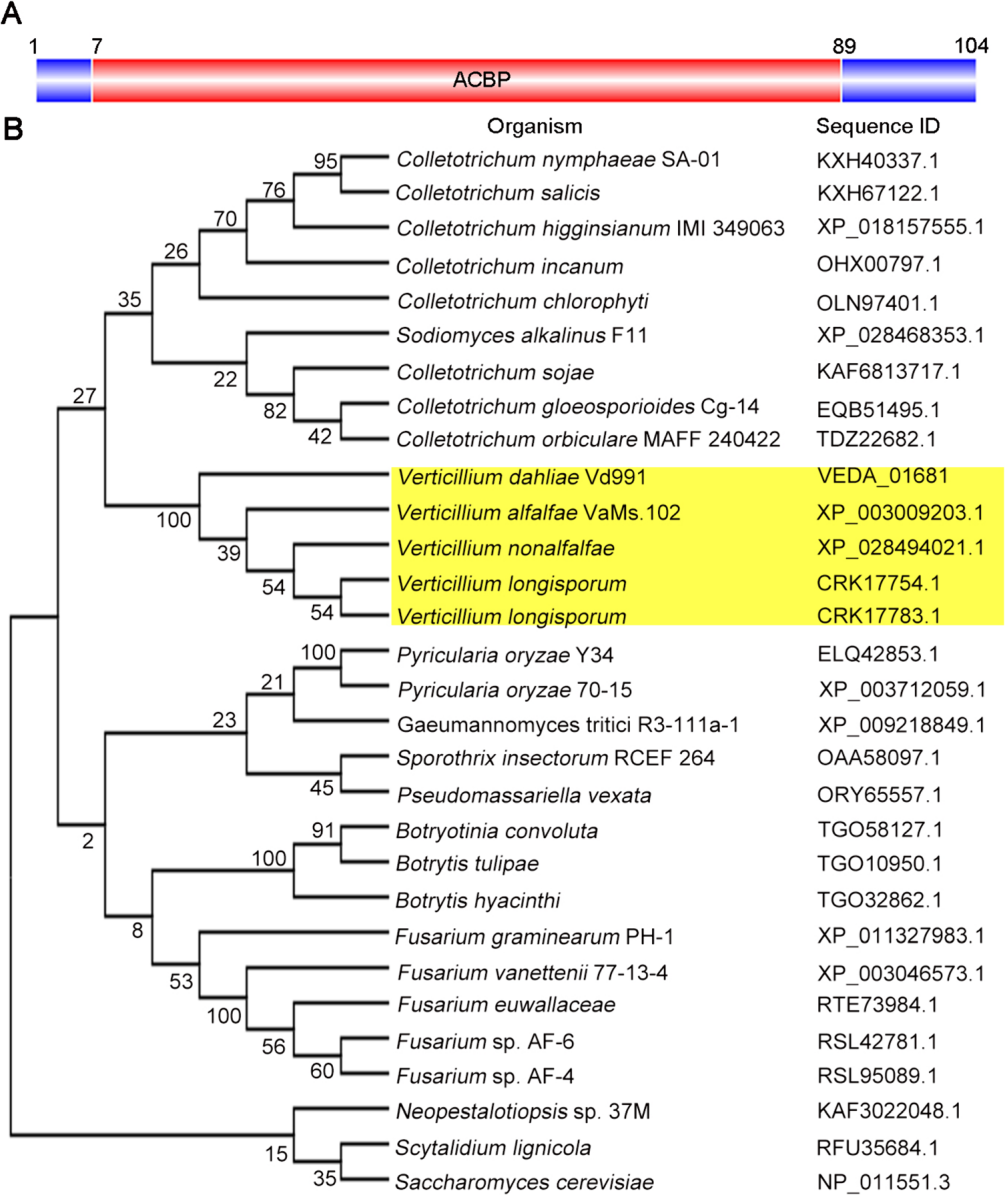
Bioinformatic analysis of the *Verticillium dahliae* VdAcb1. **A** Structure of the VdAcb1 protein as determined in a conserved domain search of NCBI. ACBP, acyl-CoA-binding protein domain. Numbers indicate amino acid positions demarcating the signal peptide region and conserved acyl-CoA-binding protein (ACBP) domain. **B** Phylogenetic analysis of ACBPs from *V. dahliae* and other fungi in the Ascomycotina. Yellow box represents the genus *Verticillium.* Sequence IDs represent the GenBank (https://www.ncbi.nlm.nih.gov/genbank/) accession number of the different acyl-CoA-binding proteins in Ascomycotina

Exoproteome analysis of *V. dahliae* Vd991 induced by cotton medium identified a putative acyl-CoA-binding protein (VEDA_01681, Gene-ID in VdLs.17 genome: VDAG_00497). Based upon the conservation of amino acid residues in this protein and in the acyl-CoA-binding domain of this protein with other fungal ACBPs (Fig. S1A), some of which have been structurally and functionally characterized (Teilum et al. 2005), the protein was designated as VdAcb1. CD-Search of the NCBI revealed that VdAcb1 has an acyl-CoA-binding protein domain (ACBP, pfam00887, from S-6 to V-84) of 83 amino acids (Fig. 1A). The *VdAcb1* CDS encodes a protein of 104 amino acids with a relative molecular weight of 11.29 kDa and an isoelectric point of 5.15 (http://www.expasy.org), consistent with the size of ACBPs of other species (Teilum et al. 2005). The 545 bp *VdAcb1* gene contains three exons of 98, 71 and 143 bp and two introns of 156 and 81 bp (Fig. S1A). To further examine the evolutionary relationship of VdAcb1 in different fungi, BLASTp was performed at NCBI, along with sequence alignment, and phylogenetic tree construction with the VdAcb1 orthologs from Ascomycotina. The phylogeny revealed that VdAcb1 shared 98% identity to *Verticillium* spp and 70% to *Colletotrichum* spp, but was more distantly related to *Pyricularia* spp. and *Fusarium* spp, with 61% and 58% identities, respectively (Fig. 1B and S1B). VdAcb1 was previously detected in the exoproteome of *V. dahliae* (Chen et al. 2016), suggesting that VdAcb1 can be secreted. However, SignalP predictions resulted in no evidence of a signal peptide at the *N*-terminus of VdAcb1 (with scores of 0.9995, above the threshold of 0.5) (Fig. S1C). Furthermore, the SecretomeP 2.0 prediction suggested that VdAcb1 may be an unconventional secreted protein (with SecP scores of 0.937, above the threshold of 0.5) (Fig. S1D). These results suggested that *VdAcb1* encodes a typical acyl-CoA-binding protein and the unconventional secreted protein was also conserved in the Ascomycotina.

### VdAcb1 is an unconventional secreted protein and its secretion is dependent on VdGRASP

**Fig. 2:**
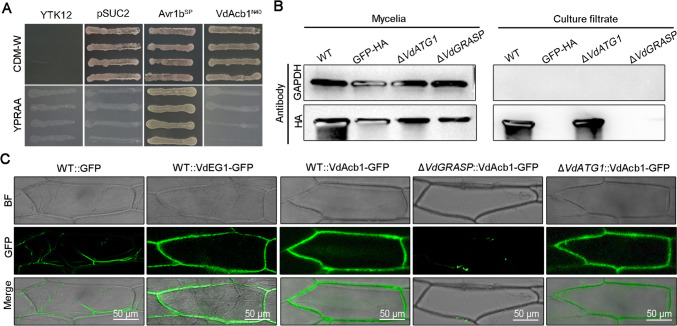
VdAcb1 is an unconventionally secreted protein and its secretion depends on VdGRASP in *Verticillium dahliae*. **A** Analysis of the function of *N*-terminal peptide of VdAcb1 by a yeast signal trap assay. The sequence of the 40 aa *N*-terminal of VdAcb1 was fused in frame to the invertase sequence in the vector pSUC2 and transformed into yeast strain YTK12. The YTK12 stain and YTK12 with empty pSUC2 vector were used as the negative controls. The YTK12 strain carrying the pSUC2 vector fused with the signal peptide of the oomycete effector Avr1b was used as the positive control. Four yeast strains were cultured on YPRAA and CMD-W media. **B** Detection of VdAcb1-HA in both mycelia and cultural filtrate of wild type (Vd991), Δ*VdGRASP,* and the Δ*VdATG1* strains*.* The mycelia and culture filtrate proteins of the indicated strains were extracted and immunoblot analyses were carried out using anti-HA and anti-GADPH antibodies. **C** Observation of onion epidermal cells infected with *V. dahliae* strains WT::VdAcb1-GFP, Δ*VdGRASP*::VdAcb1-GFP, Δ*VdATG1*:: VdAcb1-GFP, WT::VdEG1-GFP (positive control), WT::GFP and (negative controls), respectively. Images were taken 5 days after infection at 25 °C using a confocal microscope with an excitation wavelength of 488 nm and emission of 510 nm for GFP. Scale bar = 50 μm

To verify the absence of signal peptide activity at the *N*-terminus of VdAcb1, a yeast signal sequence trap assay was employed. The region encoding the polypeptide sequence of 40 amino acids at the *N*-terminus of VdAcb1 was fused to the pSUC2 vector, which carries an invertase gene without the signal peptide, to generate pSUC2-VdAcb1^N40^. While strain YTK12::pSUC2-VdAcb1^N40^ containing recombinant plasmids grew normally on CMD-W medium containing sucrose as the sole carbon source, YTK12::pSUC2-VdAcb1^N40^ could not secrete invertase extracellularly, and therefore could not grow properly on YPRAA medium, which contains raffinose as the only carbon source (Fig. 2A). As predicted by SignalP, the result indicated that the *N*-terminus of VdAcb1 does not possess a signal peptide for typical secretion through Golgi–ER systems.

To examine whether VdAcb1 could be secreted from *V. dahliae*, an HA-tag was ligated to the *C*-terminus of VdAcb1 and expressed in the wild-type strain Vd991. Thus, the secretion of VdAcb1 was verified by western blotting of the culture filtrate of the WT::VdAcb1-HA overexpressing strain with an anti-HA antibody (Fig. 2B). VdAcb1-HA was detected in the mycelia and culture filtrate of WT::VdAcb1-HA strain. But the control GAPDH was only found in the mycelia, and not in the culture filtrate (Fig. 2B). To further confirm whether VdAcb1 is secreted in vivo, we examined the subcellular location of VdAcb1 using an onion epidermal cell system. To this end, VdAcb1 was GFP-tagged in the wild-type background to produce the WT::VdAcb1-GFP strain. As shown in Fig. 2C, the green fluorescence of the VdAcb1-GFP fusion was mainly distributed at the onion epidermal cell walls like the positive control VdEG1, which is a secreted protein in *V. dahliae* that possesses a signal peptide (Gui et al. 2017). However, the green fluorescence of the negative control WT::GFP was randomly distributed in the mycelia of *V. dahliae.* (Fig. 2C). Together, these results demonstrate that VdAcb1 is secreted extracellularly by *V. dahliae* as an unconventional secreted protein without a signal peptide.

In *S. cerevisiae* (Bruns et al. 2011), *Pichia pastoris* (Manjithaya et al. 2010) and *Dictyostelium discoideum* (Kinseth et al. 2007), Acb1 is secreted in an ER–Golgi independent manner. In *S. cerevisiae*, the secretion of Acb1 depends on the Golgi reassembly and stacking protein (GRASP), which can form a novel membrane compartment called CUPS (compartment for unconventional protein secretion) (Bruns et al. 2011). Thus, we constructed the *VdGRASP* deletion mutant (*VdGRASP*, VEDA_09322, Gene-ID in VdLs.17 genome: VDAG_03042) to determine whether the secretion of VdAcb1 requires the participation of VdGRASP in *V. dahliae.* Furthermore, the VdAcb1-HA and VdAcb1-GFP constructs were transformed into the Δ*VdGRASP* strain, respectively. The results showed that VdAcb1-HA was not detected in the culture filtrate of Δ*VdGRASP* as compared to the wild-type (Fig. 2B). Moreover, the green fluorescence was not detected on the onion inner epidermal cell walls infected by the Δ*VdGRASP*::VdAcb1-GFP strain (Fig. 2C). Taken together with the findings of VdAcb1-HA localization, these results indicate that the unconventional secretion of VdAcb1 relies on VdGRASP.

Autophagy proteins (ATGs) are also involved in the secretion of Acb1 in *S. cerevisiae*, which can fuse with recycling endosomes to release cargo (Manjithaya et al. 2010). Thus, we also overexpressed *VdAcb1* in the Δ*VdATG1* background (*VdATG1*, VEDA_06789, Gene-ID in VdLs.17 genome: VDAG_05745). VdAcb1 was detected in the culture filtrate of Δ*VdATG1*::VdAcb1-HA (Fig. 2B). And further GFP fluorescence was also observed clearly on the onion epidermal cell walls following incubation with Δ*VdATG1*::VdAcb1-GFP and WT::VdAcb1-GFP (Fig. 2C). Therefore, the secretion pathway of VdAcb1 in *V. dahliae* is independent of VdATG1, and differentiated from that of *S. cerevisiae*. In general, we conclude that the extracellular release of VdAcb1 depends on VdGRASP but not VdATG1.

### VdAcb1 participates in the carbon starvation response of *V. dahliae*

To determine the function of VdAcb1 in *V. dahliae*, we constructed the *VdAcb1* gene deletion mutants (Δ*VdAcb1*) and complemented strains (EC). The colony morphology and diameter of the Δ*VdAcb1* did not differ from the wild-type (Vd991) on PDA and CM media (Fig. S2A and B). The number of conidia produced by different strains grown on PDA plates for 9 days revealed that the Vd991, Δ*VdAcb1*, and complemented strains (EC) produced similar amounts of conidia (Fig. S2C). Therefore, *VdAcb1* did not affect the growth of *V. dahliae* under nutrient-rich culture conditions.

**Fig. 3:**
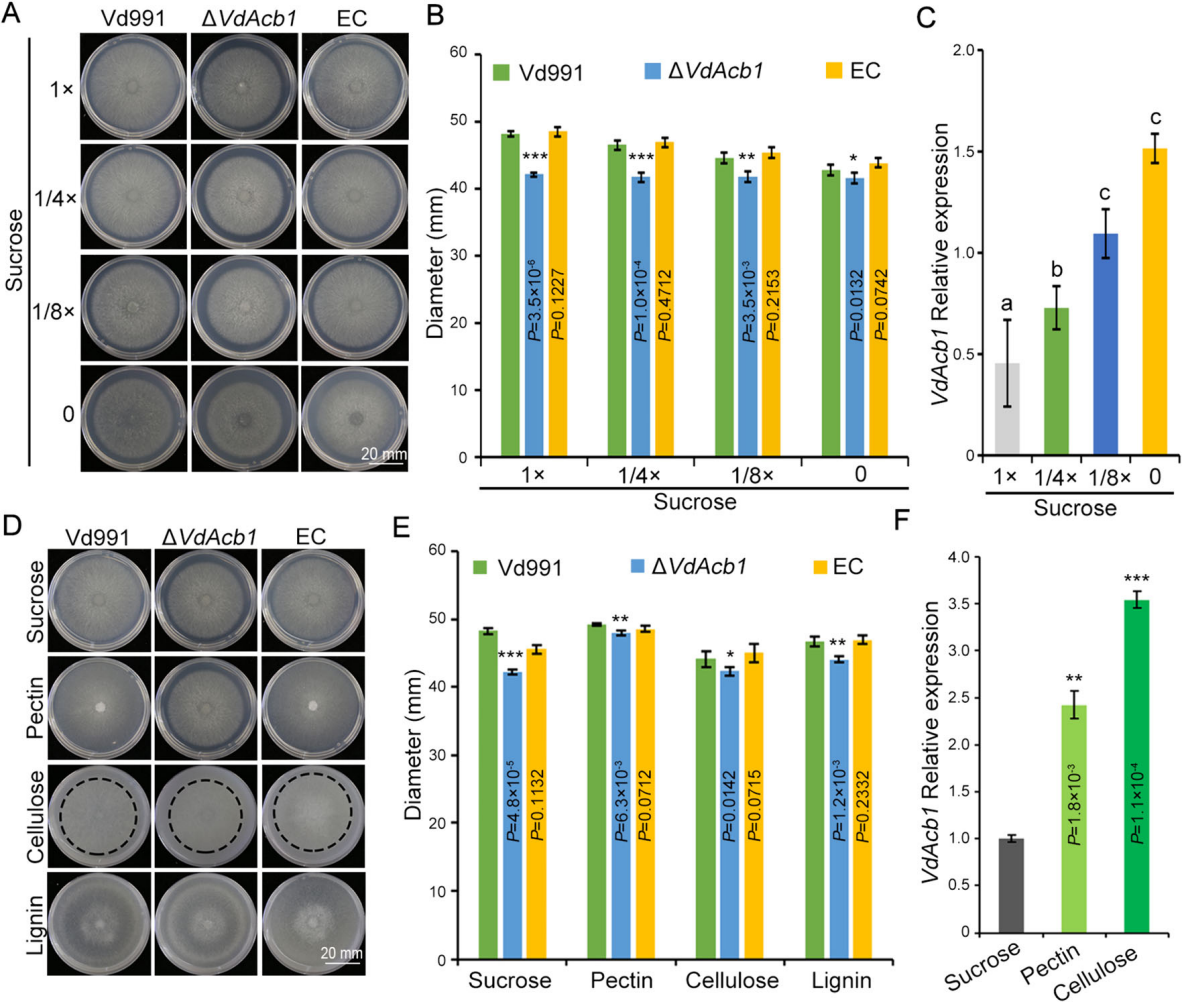
VdAcb1 positively regulates utilization of carbon sources of *Verticillium dahliae*. Radial growth (**A**) and diameter (**B**) of the wild type (Vd991), Δ*VdAcb1* and complemented (EC) strains on Czapek salt medium containing different concentrations of sucrose for 12 days at 25 °C. **C** RT-qPCR analysis of *VdAcb1* expression in Czapek salt medium containing different concentrations of sucrose. Error bars represent the standard deviation between triplicate experiments. Statistical significance was determined by one-way ANOVA with Tukey’s multiple comparisons test. Growth phenotype (**D**) and diameter (**E**) of Vd991, Δ*VdAcb1*, and complemented (EC) strains were analyzed on Czapek salt medium supplemented with sucrose (30 g/L), pectin (10 g/L), cellulose (10 g/L) and lignin (10 g/L) as carbon sources for 12 days at 25 °C. **F** RT-qPCR analysis of *VdAcb1* expression in Czapek salt medium supplemented with sucrose, pectin, and cellulose as carbon sources. The statistical significance of **B**, **E** and **F** was calculated by an unpaired student *t*-test. *, ** and *** represent significance at *P* < 0.05, *P* < 0.01 and *P* < 0.001, respectively. The error bars of **B** and **E** represent the standard deviation of three biological replicates. The error bars of **F** represent the standard deviation between triplicate experiments. **A** and **D** scale bar = 20 mm

Starvation can induce the secretion of *Acb1* in *S. cerevisiae* (Cruz-Garcia et al. 2018). To examine whether the effect of *VdAcb1* on carbon source utilization is related to the starvation response of *V. dahliae*, sucrose at different concentration gradients was used to simulate the process of continuously amplifying the starvation signal of *V. dahliae*. The growth defects in the Δ*VdAcb1* strain were gradually ameliorated with decreasing sucrose concentrations (from 1 × (30 g/L), 1/4 × (7.5 g/L) to 1/8 × (3.75 g/L) sucrose), and growth was comparable between the wild-type strain and Δ*VdAcb1* strains in the zero sucrose media (Fig. 3A, B). Moreover, we analyzed the expression levels of *VdAcb1* under varying concentrations of sucrose containing Czapeck-Dox medium, finding that starvation stress induces the expression of *VdAcb1* and the transcriptional level of *VdAcb1* was highest under the sucrose-free condition (Fig. 3C). These results indicated that starvation can complement the growth defects of *ΔVdAcb1*, and demonstrate the role of *VdAcb1* in enhancing the adaptability of *V. dahliae* under nutritional stress. As *V. dahliae* colonizes plants within the nutrition-poor xylem tissue, where polysaccharides such as pectin and cellulose serve as a primary carbon source (Klosterman et al. 2011; Zhang et al. 2022), we examined the growth of wild-type (Vd991), Δ*VdAcb1,* and complemented strains (EC) on media supplemented with sucrose, pectin, cellulose, and lignin as carbon sources. The colony diameters of Δ*VdAcb1* were reduced by 13%, 3%, 4% and 6% relative to wild-type, respectively (Fig. 3D, E). The growth defect of Δ*VdAcb1* on the polysaccharide medium (pectin, cellulose and lignin) was significantly reduced compared to that of sucrose (Fig. 3D, E), a result similarly observed by reducing sucrose concentration (Fig. 3A, B). Therefore, we examined the expression of *VdAcb1* under different carbon starvation conditions in *V. dahliae*. The expression of *VdAcb1* induced by pectin and cellulose was significantly higher than that induced by sucrose (Fig. 3F). These results suggested that VdAcb1 may be involved in the response of *V. dahliae* to starvation during infection.

### RNA-seq revealed that *VdAcb1* could regulate the expression of starvation response-related genes

**Fig. 4:**
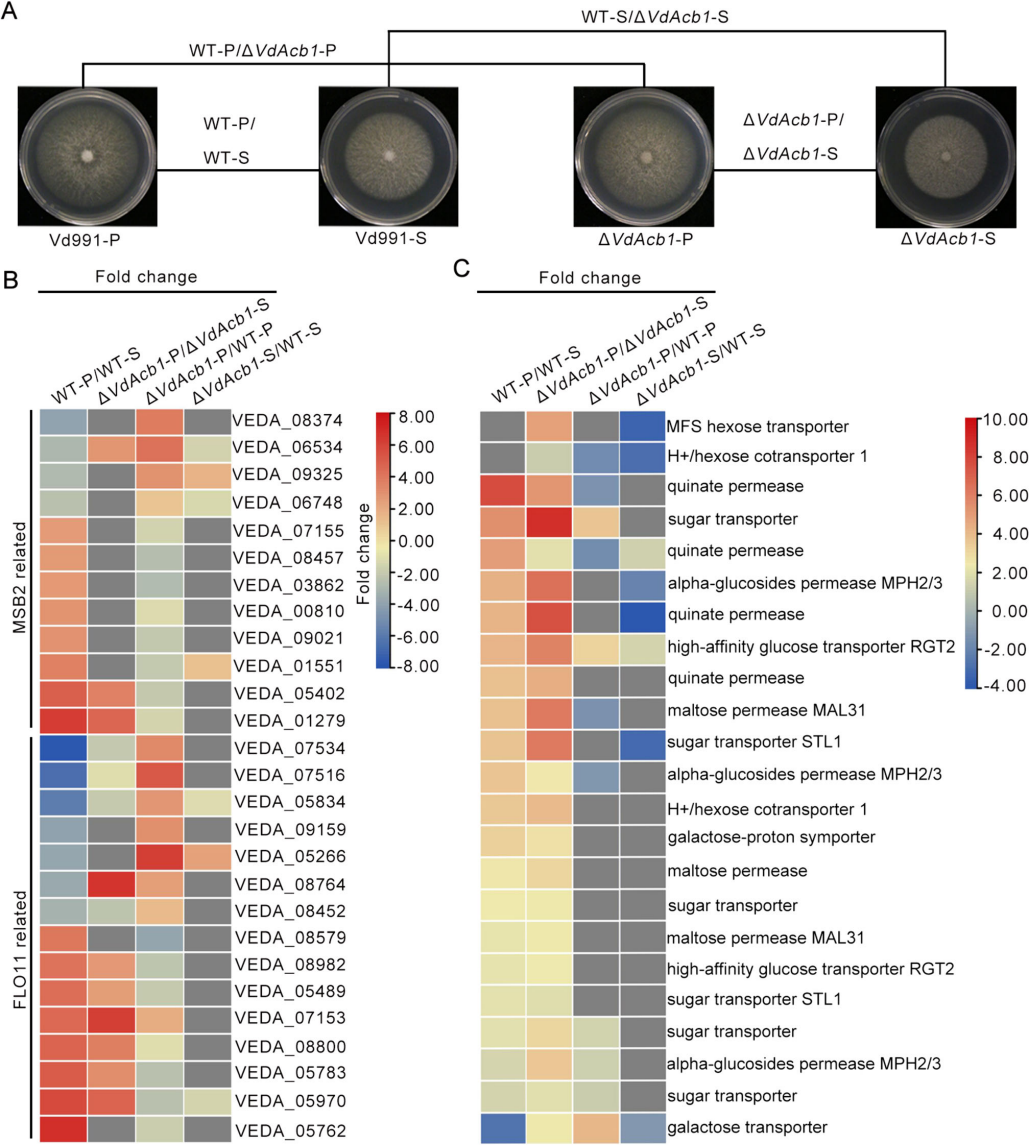
VdAcb1 regulates the expression of starvation response genes in *Verticillium dahliae*. **A** Analysis of differential gene expression (DEGs) in *VdAcb1* regulatory region by comparing expression of the wild-type grown on pectin medium (WT-P) versus wild type grown on sucrose medium (WT-S), the Δ*VdAcb1* strain grown on pectin medium (Δ*VdAcb1*-P) versus the Δ*VdAcb1* strain grown on sucrose medium (Δ*VdAcb1*-S), Δ*VdAcb1*-S vs WT-S and Δ*VdAcb1*-P vs WT-P. All samples were collected after 5 days of incubation at 25 °C. **B** Fold-change of differentially expressed *MSB2*-related genes and *FLO11*-related genes in the filamentous growth pathway. The gene IDs in Vd991 are shown on the right side of the colored blocks. The fold-change represents the value of the log_2_ ratio. **C** Expression pattern of sugar transport-related proteins in four comparison groups. The annotations corresponding to each group of color blocks are shown on the right. The gene ID corresponding to the sugar transport-related proteins are shown in Table S2. The fold change represents the value of the log_2_ratio

The defective growth of the Δ*VdAcb1* strain under starvation conditions with polysaccharides as carbon sources was less than that under nutrient-rich media with sucrose as carbon source (Fig. 3D,E). Therefore, we compared differentially expression of genes (DEGs) in the Vd991 and Δ*VdAcb1* strains treated with pectin and sucrose, respectively (Fig. 4A**)**. *MSB2*, a signaling mucin, can induce the expression of cell-adhesion flocculin *Flo11* through the MAPK pathway to regulate the filamentous growth (FG) in fungi (Cullen and Sprague 2012). In the wild-type strain Vd991, the FG pathway, including the *MSB2*-related genes and *FLO11*-related genes, were responsive to starvation (pectin) treatment (WT-P vs WT-S), but under the same conditions, these genes in Δ*VdAcb1* showed the opposite trend in their expression (Δ*VdAcb1*-P vs WT-P) (Fig. 4B). This suggested that VdAcb1 can positively regulate the FG pathway in relation to the starvation response of *V. dahliae*.

Pathogens can use sugar transporters to access nutrients from hosts (Chen et al. 2010; Sutton et al. 2007). Among the DEGs, we also found that some genes related to sugar transport (gene ID in Table S2) changed regularly under induction conditions (Fig. 4C). These genes were up-regulated in the wild-type strain Vd991 in response to pectin treatment as compared to the sucrose treatment (WT-P vs WT-S), but down-regulated in Δ*VdAcb1* strain in response to pectin compared to the wild-type strain (Δ*VdAcb1*-P vs WT-P). This indicated that the starvation conditions examined could improve the sugar transport capacity of *V. dahliae* for energy metabolism; however, the sugar transport-related genes in the Δ*VdAcb1* strain were down-regulated compared to the wild-type, even in response to the same carbon sources (Δ*VdAcb1*-P vs WT-P and Δ*VdAcb1*-S vs WT-S). Thus, VdAcb1 affects sugar transport processes in *V. dahliae*. In the summary, VdAcb1 can regulate the filamentous growth and sugar transport capacity during the starvation response by regulating the expression of starvation response-related genes.

### VdAcb1 participates in the carbon starvation through the VdMsb2 pathway

**Fig. 5:**
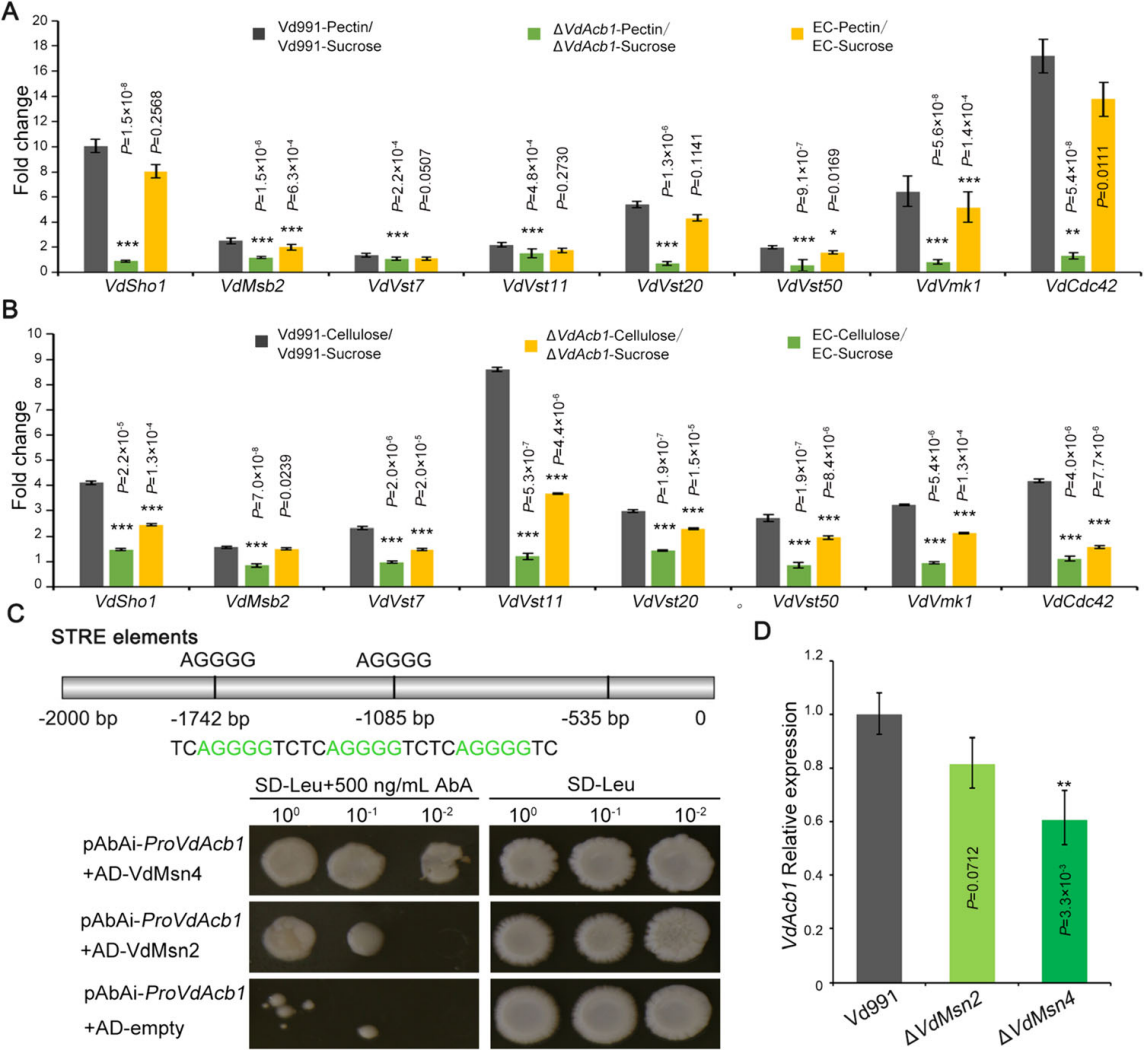
The *Verticillium dahliae* VdAcb1 participates in the carbon starvation responses through the VdMsb2 pathway. Expression analysis of *VdMsb2*-pathway genes in pectin (**A**) and cellulose medium (**B**). Wild-type (Vd991) and Δ*VdAcb1* strains were cultured on Czapek salt medium supplemented with sucrose and polysaccharide (pectin or cellulose) for 4 days. RT-qPCR analysis of the relative expression of *VdMsb2*-pathway genes in different strains induced by polysaccharide (pectin or cellulose) compared to that induced by sucrose. *VdEF-1α* was used as an endogenous control for gene expression analysis. The statistical significance of **A** and **B** was calculated by an unpaired student *t*-test. *, ** and *** represent significance at *P* < 0.05, *P* < 0.01 and *P* < 0.001, respectively. **C** Yeast one-hybrid assay of VdMsn4 binding to the *VdAcb1* promoter. The STRE elements of *VdAcb1* promoter (Top). The yeast strains containing gene-pGADT7 (VdMsn2 and VdMsn4) vector and pAbAi vector containing VdAcb1 promoter were cultured on the MDO (SD–Leu) with 500 ng/mL AbA (Below). Growth can be observed in those strains with positive interactions. The pGADT7-empty vector was used as a negative control. **D** The expression of *VdAcb1* in the wild type Vd991, Δ*VdMsn2* and Δ*VdMsn4* strains was detected under starvation induction conditions (Czapek salt medium). The expression level of *VdAcb1* in the Vd991 was set to 1 and *VdEF-1α* was used as an endogenous control for gene expression analysis. Statistical significance was calculated by an unpaired student *t*-test with ** representing significance at *P* < 0.01. The error bars of **A**, **B** and **D** represent the standard deviation between triplicate experiments

Transcriptome analysis showed that *VdAcb1* could regulate the expression of *MSB2*-pathway genes under carbon starvation (Fig. 4B). In order to further verify the results of the transcriptome, we cultured the wild type, Δ*VdAcb1*, and EC strains on the Czapek medium with sucrose or polysaccharides (pectin or cellulose) as carbon sources. In the Δ*VdAcb1* strain, the fold change in the expression of *VdMsb2* pathway genes in response to pectin and cellulose induction (Δ*VdAcb1*-pectin*/*Δ*VdAcb1*-sucrose, Δ*VdAcb1*-cellulose*/*Δ*VdAcb1*-sucrose) were significantly lower than that of the wild type (Vd991-pectin*/*Vd991-sucrose, Vd991-cellulose*/*Vd991-sucrose) (Fig. 5A, B). These results demonstrated that *VdAcb1* positively regulates the *VdMsb2* pathway in response to carbon starvation.

We further evaluated deletion mutants of *VdMsb2*-regulated FG signaling pathway, including the membrane-localized sensors VdSho1 and VdMsb2, and the MAPK pathway adaptor Vst50. The growth diameter of each of these mutants on different carbon sources examined was significantly smaller than that of wild type (Fig. S3). These results suggested that the *VdMsb2*-involved FG signaling pathway can affect the growth of *V. dahliae* under starvation conditions. In *S. cerevisiae*, starvation can induce the expression and secretion of Acb1 (Cruz-Garcia et al. 2018). To explore whether VdAcb1 is regulated by the *VdMsb2*-involved FG signaling pathway under starvation conditions, the relative expression levels of *VdAcb1* were examined in the above deletion mutant strains. The expression of *VdAcb1* in the Δ*VdSho1*, Δ*VdMsb2* and Δ*VdVst50* were all significantly lower than in the wild-type under the induction conditions of either pectin or carbon source starvation (Fig. S3C). In *S. cerevisiae*, transcription factors MSN2 and MSN4 bind specifically to the stress response elements (STRE) 5′-AGGGG or 5′-GGGGA (Stewart-Ornstein et al. 2013) and thus participate in a variety of emergency responses including starvation stress (Gasch et al. 2000). We found three STRE binding sites also located at − 535, − 1085 and − 1742 bp upstream of *VdAcb1* (Fig. 5C). Yeast one-hybrid (Y1H) assays showed that VdMsn2 and VdMsn4 can bind to the *VdAcb1* promoter (Fig. 5C). However, the binding of VdMsn4 was stronger than with VdMsn2 (Fig. 5C). The expression of *VdAcb1* in the different mutant strains was also examined under starvation induction conditions. The expression of *VdAcb1* was positively regulated by the transcription factor VdMsn4. Compared to Δ*VdMsn4*, VdMsn2 did not significantly regulate the expression of *VdAcb1*, probably due to the functional redundancy between VdMsn2 and VdMsn4 (Fig. 5D). In summary, when *V. dahliae* is induced by starvation, VdAcb1 is induced in its expression by transcription factors VdMsn2 and VdMsn4 and secreted extracellularly to regulate the VdMsb2-pathway. In turn, this further activates the starvation stress response in *V. dahliae.*

### VdAcb1 is required for virulence in *V. dahliae*

**Fig. 6:**
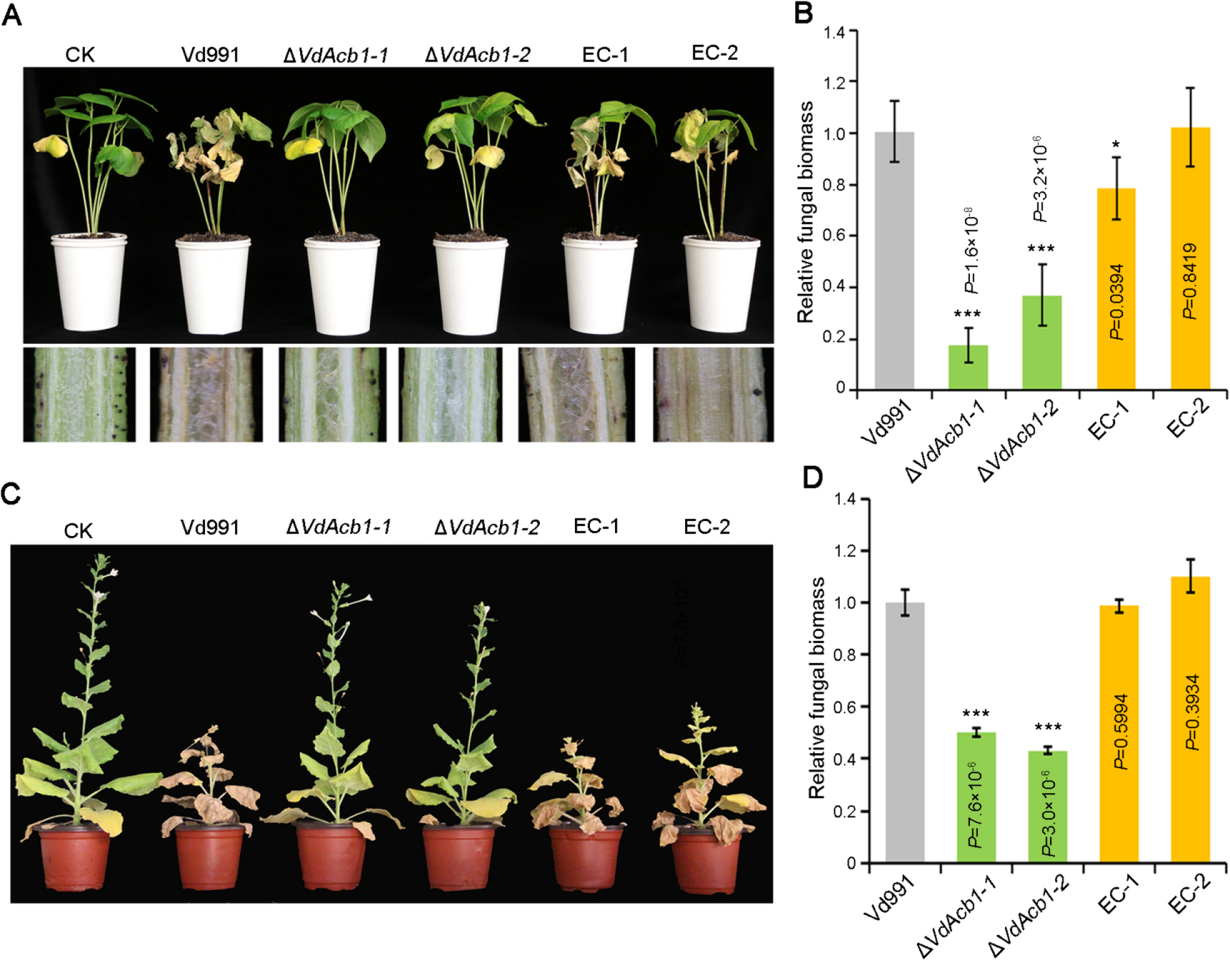
Pathogenicity analysis of *VdAcb1* deletion strains on cotton and *Nicotiana benthamiana*. **A** Disease symptoms (top) and discoloration in longitudinal sections (bottom) of cotton (*Gossypium hirsutum* cv. Junmian 1) inoculated with sterile water (CK), wild-type (Vd991), Δ*VdAcb1*, and the *VdAcb1* complemented strains (EC#1 and #2). Plants were observed 21 days after inoculation. **C** Disease phenotypes of 5-weeks-old *N. benthamiana* plants inoculated with wild-type (Vd991), Δ*VdAcb1*, and the *VdAcb1* complemented strains (EC#1 and #2). Plants were examined at 21 days after inoculation. Fungal biomass in cotton (**B**) and *N. benthamiana* (**D**) inoculated with different strains was anlayzed by qPCR. Statistical significance was calculated by an unpaired student *t*-test with * and *** representing significance at *P* < 0.05 and *P* < 0.001, respectively. Error bars represent the standard deviation between triplicate experiments

To investigate the role of VdAcb1 during the *V. dahliae* infection, we first examined the expression of *VdAcb1* in cotton roots in a series of incubation times by RT-qPCR. This revealed that the expression of *VdAcb1* was up-regulated during infection and reached the maximum at 3 days post-inoculation (Fig. S4A). To determine whether VdAcb1 plays a role in the initial penetration phase of *V. dahliae*, we analyzed the penetration ability on cellophane membranes. The penetration ability of the Δ*VdAcb1* strain was not affected at 4 days, compared to the wild-type strain (Fig. S4B). Additionally, cotton (*Gossypium hirsutum* cv. Junmian 1) and tobacco (*Nicotiana benthamiana*) were inoculated with spore suspensions of wild type, Δ*VdAcb1* and *VdAcb1*-complemented strains at the same concentration. The typical symptoms of Verticillium wilt caused by the wild-type strain Vd991, such as the yellowing of plant leaves, were reduced in plants inoculated with the Δ*VdAcb1* strain (Fig. 6A, C), and correspondingly, the browning of cotton vascular tissue was also reduced (Fig. 6A). The qPCR assays of fungal biomass in cotton and tobacco revealed that the fungal biomass was significantly lower in plants inoculated with the Δ*VdAcb1* strain than in the plants inoculated with the wild-type strain (Fig. 6B, C). In conclusion, VdAcb1 is an important virulence factor in *V. dahliae*.

## Discussion

Although acetyl-CoA binding protein is one of the most studied unconventional secreted proteins in eukaryotes (Charmpilas et al. 2020), its extracellular function in plant pathogenic fungi has not been elucidated. In this study, we found that VdAcb1 was induced by transcription factor VdMsn4 under starvation conditions and secreted into the extracellular space by an unconventional secretion. We also demonstrated that it can regulate the carbon starvation response during *V. dahliae*-plant interactions by VdMsb2-related MAPK pathway to enhance the downstream filamentous growth. VdAcb1 enhances the nutritional utilization ability of *V. dahliae* and is also a critical virulence factor as demonstrated on two host plants.

Secreted proteins are usually defined as a class of proteins with a signal peptide that can be secreted extracellularly through the ER–Golgi pathway. However, proteomics has revealed that there are a large number of proteins in fungal secretomes that lack signal peptides (González-Fernández et al. 2015; Rampitsch et al. 2013). Unconventionally-secreted proteins in plant pathogens are involved in pathogenic processes in a variety of ways. For example, *Magnaporthe oryzae* oxysterol-binding protein-related proteins (MoORPs) are detected in intercellular fluids of barley plants following *M. oryzae* infection (Chen et al. 2022). MoORPs act as PAMP molecules to regulate plant innate immunity and promote the virulence of *M. oryzae* (Chen et al. 2022). VdSOD1 and VdTrx1 lack signal peptides yet are important in scavenging intracellular and extracellular reactive oxygen species in *V. dahliae* (Tian et al. 2021, 2023). In this study, we determined that VdAcb1 lacks a typical signal peptide and the 40 amino acids at its *N*-terminus also do not have the secretory activity since this sequence failed to direct the secretion of sucrose invertase to extracellular space in the yeast signal sequence trap system (Fig. 2A). The secretion of VdAcb1 was detected in the culture supernatant of *V. dahliae* and green fluorescence was observed on the onion cell walls after co-incubation of onion cells and the *V. dahliae* strain overexpressing the *VdAcb1-GFP* fusion (Fig. 2B, C). Taken together, these results provide concrete evidence that VdAcb1 is an unconventionally secretion protein.

Studying the function of unconventionally secreted proteins in plant—microbe interactions is an emerging topic (Miura and Ueda 2018). The peroxisome protein, sterol carrier protein 2 (Scp2), was detected in the extracellular fluid of infected maize and may play a role in inhibiting the competitors in the extracellular fluid (Krombach et al. 2018). Our previous work showed that the exoproteome of *V. dahliae* strain Vd991 contains 99 proteins without signal peptides, suggesting that many more unconventionally secreted proteins probably play important roles in host–pathogen interactions (Chen et al. 2016; Wang et al. 2018a). The research on the pathogenic function of unconventionally secreted proteins in *V. dahliae* is still in the initial stages. For example, VdIsc1 can suppress salicylate-mediated innate immunity *in planta* by hydrolyzing isochorismate, a precursor of salicylate (Liu et al. 2014). VdSOD1 and VdTrx1, without signal peptides, can promote *V. dahliae* infection by scavenging reactive oxygen species (Tian et al. 2021, 2023). In this study, we further demonstrated the importance of the unconventionally secreted protein VdAcb1 during *V. dahliae* infection, and its role in responding to host carbon starvation (Fig. 3D, E) is likely critically important for its contribution to virulence (Fig. 6A, C). This work further sheds light on unconventionally secreted proteins in plant-pathogen interactions and additional focus on these proteins in plant-pathogen interactions may be fruitful.

Research on molecular mechanisms of the unconventional secretory pathway is still in its infancy. The unconventional secretory pathway is commonly categorized into four types (Rabouille et al. 2012). Among these, type III secretion is more studied in fungi. In type III unconventional protein secretion, the cargo can be integrated into the lumen of the cell inner membrane, and when fused with the plasma membrane, it mediates the transport of the cargo to the extracellular space. The membrane compartment called CUPS (compartment for unconventional protein secretion) is marked by Grh1 (homologous proteins of GRASP) in *S. cerevesiae* (Bruns et al. 2011). Acb1 and SOD1 of *S. cerevisiae* lacking signal peptides can be captured into CUPS under starvation and released into the extracellular space (Cruz-Garcia et al. 2017). However, some of the mechanisms of unconventional secretion of Acb1 can be differentiated among fungi. For example, in *S. cerevisiae*, *P. pastoris* and *Cryptococcus neoformans*, the secretion of Acb1 is not only affected by GRASP, but also regulated by autophagy-related genes (Duran et al. 2010; Manjithaya et al. 2010; Xu et al. 2016). In *Aspergillus oryzae*, the secretion of AoAcb1 is dependent on t-SNARE AoSso1, but independent of the autophagy-related protein AoAtg1 (Kwon et al. 2017). In this research, we observed the secretion efficiency of VdAcb1 in *VdGRASP* and *VdATG1* deletion strains by combining in vivo live-cell imaging and in vitro western blots. The secretion of VdAcb1 in *V. dahliae* was regulated by VdGRASP but not VdATG1 (Fig. 2B, C). In addition, our previous research had shown that the unconventional secretion of VdSOD1 requires the participation of VdGRASP and the secretion of VdTrx1 is affected by VdVps36 (Tian et al. 2021, 2023). Therefore, *V. dahliae* also secretes some proteins important for pathogenicity by unconventional secretion, and the types of unconventional secretion may also be differentiated based on the protein types.

Acyl-CoA-binding proteins represent an evolutionarily conserved protein family with an acyl-CoA binding domain, which has high specificity and affinity for long-chain fatty acyl-CoA esters (LCACoAs) (Neess et al. 2015). The reports on the function of Acb1 in fungi mainly focus on growth and development. In *Schizosaccharomyces pombe*, Acb1 is essential for maintaining mitochondrial tubular morphology, mitochondrial respiration, and remodeling lipid droplets to promote cell survival under nutrient-rich conditions (He et al. 2022). MoAcb1 is involved in conidial germination and appressorium formation in *M. oryzae* (Cao et al. 2023). However, deletion of *VdAcb1* did not affect growth of *V. dahliae* under optimal nutritional conditions (Fig. S2A, B), nor did it affect spore yield (Fig. S2C) or penetration ability (Fig. S4B). These results indicated that unlike reported functions for Acb1 in different fungi, VdAcb1 is not involved in the metabolic processes that influence a variety of functions in *V. dahliae*.

In *S. cerevisiae* and *A. oryzae*, Acb1 can be secreted into the extracellular space under carbon starvation (Cruz-Garcia et al. 2018; Kwon et al. 2017). In this study, we confirmed that VdAcb1 positively regulated the response of *V. dahliae* to carbon starvation stress when sucrose concentrations decreased or when pectin or cellulose was supplied as the carbon source (Fig. 3A–F). It is well established that *V. dahliae* colonizes the plant xylem, a nutrition-poor niche (Klosterman et al. 2011), and thus VdAcb1 may play a critical role during the colonization of this rather unique niche. Furthermore, RNA-seq analysis indicate that VdAcb1 could affect the expression of genes in the FG pathway, such as the *MSB2* and *FLO11* related genes, and also regulate the expression of sugar transport related genes (Fig. 4B, C). In fungal pathogens, filamentous growth and biofilm formation are required for virulence (Lo et al. 1997; Nobile and Mitchell 2006). Under nutrient-limited conditions, the adhesion molecule flocculin Flo11p can enhance cell–cell adhesion and cell surface properties to promote the filamentous growth of *S. cerevisiae* (Lo and Dranginis 1996). MSB2, as a cell membrane-localized signal mucin, senses a variety of external environmental changes and regulates different activities through the downstream coupling of MAPK signaling pathways (Carraway et al. 2003) including starvation signaling (Abdullah and Cullen 2009). We examined the expression levels of *VdMsb2*-related MAPK pathway genes under pectin and cellulose simulated starvation conditions and found that the expression levels of these genes were significantly reduced under starvation induction in the Δ*VdAcb1* strain (Fig. 5A, B). Thus, VdAcb1 can positively regulate the growth of *V. dahliae* in a carbon-starved environment through the *VdMsb2*-related MAPK pathway. But how does VdAcb1 regulate this pathway? Since VdAcb1 is a secreted protein, we speculate whether there will be an interaction between VdAcb1 and cell membrane-localized signal mucin to regulate the starvation response, which needs to be further verified.

**Fig. 7:**
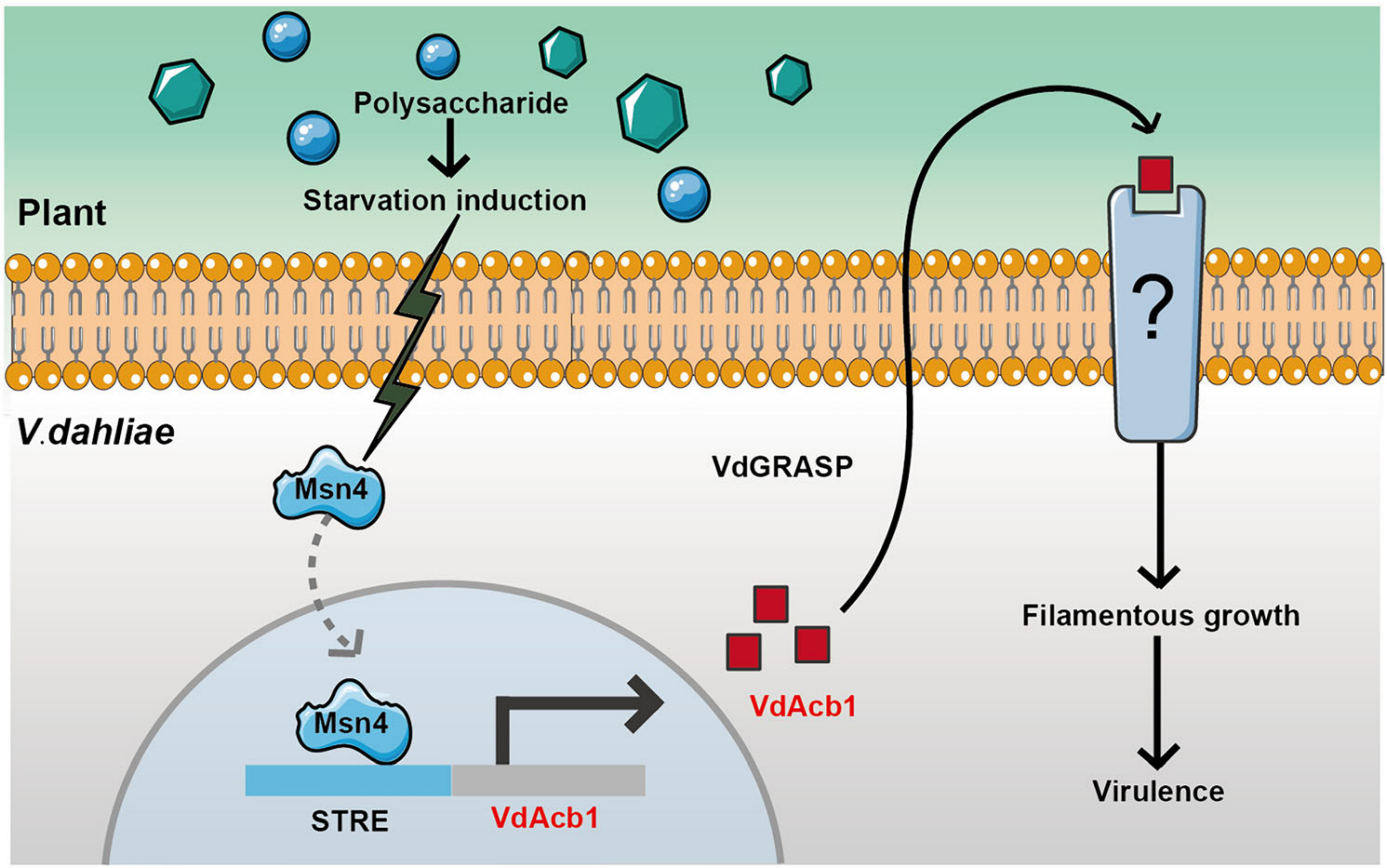
Proposed model of VdAcb1 function during *Verticillium dahliae*-host interactions. Under the carbon starvation induction, the expression of *VdAcb1* is induced by VdMsn4. VdAcb1 is then secreted extracellularly, under the guidance of VdGRASP, and it can then regulate the filamentous growth by the VdMsb2 pathway to enhance the virulence of *V. dahliae*

The activated/repressed genes specific to certain stress conditions in organisms are known collectively as “the environmental stress response genes” (ESR) (Garcia-Gimeno and Struhl 2000). The coordinated activation of hundreds of induced ESR (iESR) genes is regulated by two functionally redundant transcription factors Msn2 and Msn4 (Berry and Gasch 2008). We identified three STRE binding sites in the promoter of *VdAcb1* that were bound by VdMsn2 and VdMsn4 (Fig. 5C). The Y1H analysis in this study further confirmed that VdMsn4 could directly bind to the promoter of *VdAcb1* (Fig. 5C). Previous research also determined that the subcellular localization of Msn2 and Msn4 is regulated by the availability of carbon sources: if cells are grown in glucose, Msn2/4 is mainly located in the cytoplasm, but if grown in carbon-deficient medium, Msn2/4 is mainly located in the nucleus (Mayordomo et al. 2002). Once entering the nucleus, Msn2/4 regulates the expression of genes related to carbon source utilization such as a UDP-glucose pyrophosphorylase (Ugp1) (Yi and Huh 2015). Moreover, Msn2/4 were found to be regulated by the major signaling pathways including the cAMP-PKA to respond to starvation (Garmendia-Torres et al. 2007; Lee et al. 2008). In this study, we also observed that VdMsn4 can promote the expression of *VdAcb1* (Fig. 5D). This further supports a role of VdAcb1 in carbon source utilization. Thereafter, VdAcb1 is secreted extracellularly via an unconventional secretion mechanism. Then it can promote theVdMsb2-related MAPK pathway to enhance the starvation response during infection (Fig. 7). Therefore, we speculate that VdAcb1 acts to link the starvation signaling functions of the cAMP-PKA and VdMsb2-related MAPK pathways.

In summary, *V. dahliae* faces carbon starvation stress and carbon resources dominated by complex polysaccharides such as cellulose and pectin during infection. Starvation signals can be transmitted to transcription factor VdMsn4. And the expression of *VdAcb1* can be activated by VdMsn4 and secreted extracellularly by an unconventional secretion pathway dependent on the Golgi membrane-associated protein VdGRASP. VdAcb1 is able to regulate the VdMsb2-related MAPK signaling pathway, thereby enhancing nutrient utilization and filamentous growth. Therefore, VdAcb1 is important for *V. dahliae* in adapting to a carbon starved environment during infection and thus contributes to virulence.

## Materials and methods

### Bioinformatics analysis

The DNA and cDNA sequences of *VdAcb1* were identified in the genome database of Vd991 (Chen et al. 2018). Proteins homologous to VdAcb1 were searched by BLASTp on the NCBI database. Bioedit v7.2.0 (http://www.mbio.ncsu.edu/bioedit/) was used for the sequence alignment of homologous proteins. The phylogenetic tree of VdAcb1 was prepared using neighbor-joining in MEGA 6.0 (Tamura et al. 2013). The conserved domain of VdAcb1 was predicted using SMART (Letunic et al. 2021). SignalP v. 5.0 (Almagro Armenteros et al. 2019) was used in signal peptide prediction (Almagro Armenteros et al. 2019) and SecretomeP v.2.0 (Bendtsen et al. 2005) was used to predict unconventional secretion of VdAcb1.

### Fungal strains and vector construction

All transgenic strains of *V. dahliae* in this study were constructed using an ATMT method. For the preparation of single gene knockout strains, the upstream and downstream (1.1–1.5 kb) sequences of the target genes were amplified from the wild-type Vd991 genomic DNA and integrated into *EcoR*I and *Xba*I restriction endonuclease sites of pGKO2-*Hyg* (Zhou et al. 2013), respectively. The plasmid was transferred into Vd991, and transformants were screened with 200 μg/mL cefotaxime, 50 μg/mL hygromycin, and 200 μg/mL 5-fluoro-2′-deoxyuridine. To construct the complementation vector for *VdAcb1*, the native promoter (1 kb) and terminator (0.5 kb) of the target gene was ligated to a *Xba*I-linearized pCOM vector (Zhou et al. 2013). The recombinant plasmid was transformed into the Δ*VdAcb1* strain. To obtain the overexpression transformants of *VdAcb1*, the cDNA of *VdAcb1* was inserted into the *Kpn*I site of the pCOM-GFP vector (Tian et al. 2021) and *Sac*I/*Xba*I sites of the pCOM-TrpC vector with an HA tag (Zhou et al. 2013). The recombinant plasmids were transferred into Vd991, Δ*VdGRASP* and Δ*VdATG1*. Positive overexpression strains were selected on PDA medium containing 200 μg/mL cefotaxime and 50 μg/ mL geneticin (G418). DNA of different strains was extracted and verified to contain the correct insertions by PCR. The primers used are shown in Table S1.

### Colony growth phenotype and conidiation assays

The spore concentration of the different *V. dahliae* strains was adjusted to 5 × 10^6^ conidia/mL using a hemocytometer. Two-microliters of the conidial suspension were placed on PDA and CM media and incubated at 25 °C for 9 days. Different carbon sources (sucrose 30 g/L, pectin 10 g/L, cellulose 10 g/L, lignin 10 g/L, glucose 10 g/L and fructose 10 g/L) and sucrose of different concentrations as 1 × (30 g/L), 1/4 × (7.5 g/L), 1/8 × (3.75 g/L) and zero in Czapek salts (NaNO_3_ 3 g/L, MgSO_4_⋅7H_2_O 0.5 g/L, KCl 0.5 g/L, FeSO_4_⋅7H_2_O 0.01 g/L, K_2_HPO_4_ 1 g/L, Agar 18 g/L) were used for carbon source utilization analysis. The colony diameters were measured at 9 days.

To analyse the conidiation*,* the wild type, Δ*VdAcb1*, and complementary strains were cultured on PDA at 25 °C. Three blocks of mycelia were cut out from the edge of a 9 day-old colony by using the 5 mm-diameter puncher. After shaking in sterile water for 1 min, the number of conidia was counted using a hemocytometer.

### Yeast signal sequence trap system and infection of onion epidermal cells

Functional validation of the predicted signal peptide was performed as described previously (Jacobs et al. 1997). The *N*-terminal sequence of *VdAcb1* was fused into the pSUC2 vector at the *EcoR*I and *Xho*I sites. The recombinant plasmid (VdAcb1^N40^) was transformed into yeast competent YTK12 and cultured on CMD-W (lacking tryptophan) medium. The yeast strain YTK12 with functional signal peptide of Avr1b was the positive control. The YTK12 strain and transformants with pSUC2 alone were negative controls. The growth of these strains on YPRAA (2% raffinose) medium was used to assay activity of the signal peptide.

The spore suspensions of the GFP-tagged VdAcb1 overexpressing strains (WT::VdAcb1-GFP, Δ*VdGRASP*::VdAcb1-GFP, Δ*VdATG1*::VdAcb1-GFP) were adjusted to 1 × 10^7^ conidia/mL. The strains with GFP and fused proteins (VdEG1-GFP) were the negative and positive controls, respectively. The onion inner epidermis was immersed in the respective spore suspensions for 30 min. The onion inner epidermis was overlayed on 1% water agar for 5 days at 25 °C. GFP fluorescence in the epidermal cells was observed by laser confocal microscopy (ZEISS, LSM 880) at emission and excitation wavelengths of 488 and 510 nm, respectively.

### Protein extraction and western blot

The HA-tagged VdAcb1 overexpressing strains of *V. dahliae* (WT::VdAcb1-HA, Δ*VdGRASP*::VdAcb1-HA, Δ*VdATG1*::VdAcb1-HA) were grown in CM liquid medium for 4 days at 25 °C. Total proteins were extracted from the collected mycelia by using RIPA lysate (Beyotime, P0013K) with 1 mM phenylmethylsulfonyl fluoride (PMSF) (Solarbio, P0100). To extract the secreted proteins, the supernatant of each of the respective strains was mixed with phenol, and the secreted proteins were precipitated with methanol containing 100 mM ammonium acetate. The secreted proteins were collected following centrifugation at 4000 rpm for 10 min.

To detect protein secretion, SDS–PAGE (10% separation gel and 5% concentrated gel) was prepared for western blotting. Proteins were transferred to Immobilon-P transfer membranes (Merck Millipore) at 110 V for 1 h. The membrane was blocked in 5% (w/v) nonfat dry milk for 1 h. The membrane was incubated with anti-HA (Abmart, M20003, 1:5000) and anti-GAPDH (Abclonal, AC035, 1:5000) primary antibody for 1 h. The secondary goat anti-mouse IgG-HRP (TransGen Biotech, HS201-01, 1:5000) was used for detection with an Immobilon Western Chemiluminescent HRP Substrate (Merck Millipore).

### RNA extraction and RT-qPCR

To detect expression of *VdAcb1* during the cotton root infection process, total RNA was extracted from cotton roots collected at 0 day, 1 day, 3 days, 5 days, 7 days, and 9 days after inoculations with Vd991 using a total RNA Miniprep kit (Aidlab). To detect the function of VdAcb1 in the starvation pathway, different strains were cultured in Czapek liquid medium (NaNO_3_, 3 g/L; K_2_HPO_4_, 1 g/L; MgSO_4_⋅7H_2_O, 0.5 g/L; KCl, 0.5 g/L; FeSO_4_, 0.01 g/L) with sucrose (10 g/L), pectin (10 g/L) and cellulose (10 g/L) as carbon sources and Czapek salt (no carbon sources) for 4 days at 25 °C. RNA was extracted from the collected mycelia.

The cDNA was reverse transcribed using TransScript II One-Step gDNA Removal and cDNA Synthesis SuperMix (TransGen Biotech). The qPCR was carried out using 2 × Taq Pro Universal SYBR qPCR Master Mix (Vazyme). The amplification reaction process included pre-denaturation at 95 °C for 10 min, followed by 40 cycles of 95 °C denaturation for 15 s, 60 °C annealing for 30 s, and 72 °C extension for 30 s. The expression of *VdAcb1* at different infection times was detected by using the cotton 18S rRNA (*Gh18S*) as the internal reference gene. The *V. dahliae VdEF-1α* gene was used as endogenous reference gene to examine the expression of genes related to starvation pathways. The relative transcript levels of the above genes were analyzed by the 2^−∆∆Ct^ method (Livak and Schmittgen 2001). Primers for the RT-qPCR expression analyses are listed in Table S1.

### RNA-Seq

The wild-type and Δ*VdAcb1* strains of *V. dahliae* were cultured in Czapek medium containing sucrose (10 g/L) and pectin (10 g/L) respectively for 4 days at 25 °C. Total RNA was extracted for RNA-seq using total RNA Miniprep kit (Aidlab, RN38). Transcriptome data analyses were conducted as previously described (Zhang et al. 2018).

### Penetration and pathogenicity analysis

The conidial densities of the wild-type, Δ*VdAcb1*, and *VdAcb1-*complemented (EC) strains were adjusted to 1 × 10^7^ conidia/mL using a hemocytometer. The spore suspensions were incubated on MM with a sterilized cellophane membrane. The respective strains were cultured for 4 days on MM at 25 °C, the membrane was removed, and the MM plates were incubated for four additional days at 25 °C.

Evaluation of strain virulence was made according to the method described by (Sun et al. 2021). Three-week-old cotton plants (*Gossypium hirsutum* cv. Junmian 1) were immersed in the spore suspensions (1 × 10^7^ conidia/mL) of the different strains for 30 min. The inoculated cotton was replanted in the soil. Four-week-old tobacco (*N. benthamiana*) plants were inoculated by root-irrigation (Zhang et al. 2019). For each strain within each experiment, 20 cotton plants or five tobacco plants were inoculated. The disease symptoms on cotton and tobacco were observed 21 days after inoculation and the vascular discoloration in cotton root and stem was scored. The inoculation experiments were repeated once.

For analyses of fungal biomass, DNA was extracted from the root–stem junction of cotton and tobacco plants 21 days after inoculation. The *V. dahliae EF-1α* (*VdEF-1α*) was quantified by qPCR using the cotton *18S rRNA* (*Gh18S*), and *N. benthamiana EF-1α* (*NbEF-1α*) as internal reference genes. The amplification reaction process included pre-denaturation at 95 °C for 10 min, followed by 40 cycles of 95 °C denaturation for 15 s, 60 °C annealing for 30 s, and 72 °C extension for 30 s. The primers used are listed in Table S1.

### Yeast one-hybrid

Yeast one-hybrid assays the replicated STRE element (AGGGG × 3) was ligated into the pAbAi vector and the respective CDS of each gene (*VdMsn2* and *VdMsn4*) was cloned into the pGADT7 vector. Recombinant plasmids were co-transformed into Y1HGold. Detection of gene activation by transcription factors was performed using the MDO (SD–Leu) medium with 500 ng/mL AbA.

## Supplementary Information

Below is the link to the electronic supplementary material.

Supplementary file1 (DOCX 1764 KB)

## Data Availability

The data that support the findings of this study are available from the corresponding author upon reasonable request.
