# Supplementary material for: The acyl-CoA-binding protein VdAcb1 is essential for carbon starvation response and contributes to virulence in Verticillium dahliae
Source: aBIOTECH. 2024 Jul 13;5(4):431–48. doi: 10.1007/s42994-024-00175-3 (PMC11624172; doi:10.1007/s42994-024-00175-3)
Supplement: Supplementary file 1 — Supplementary file1 (DOCX 1764 KB) [file 42994_2024_175_MOESM1_ESM.docx]

**
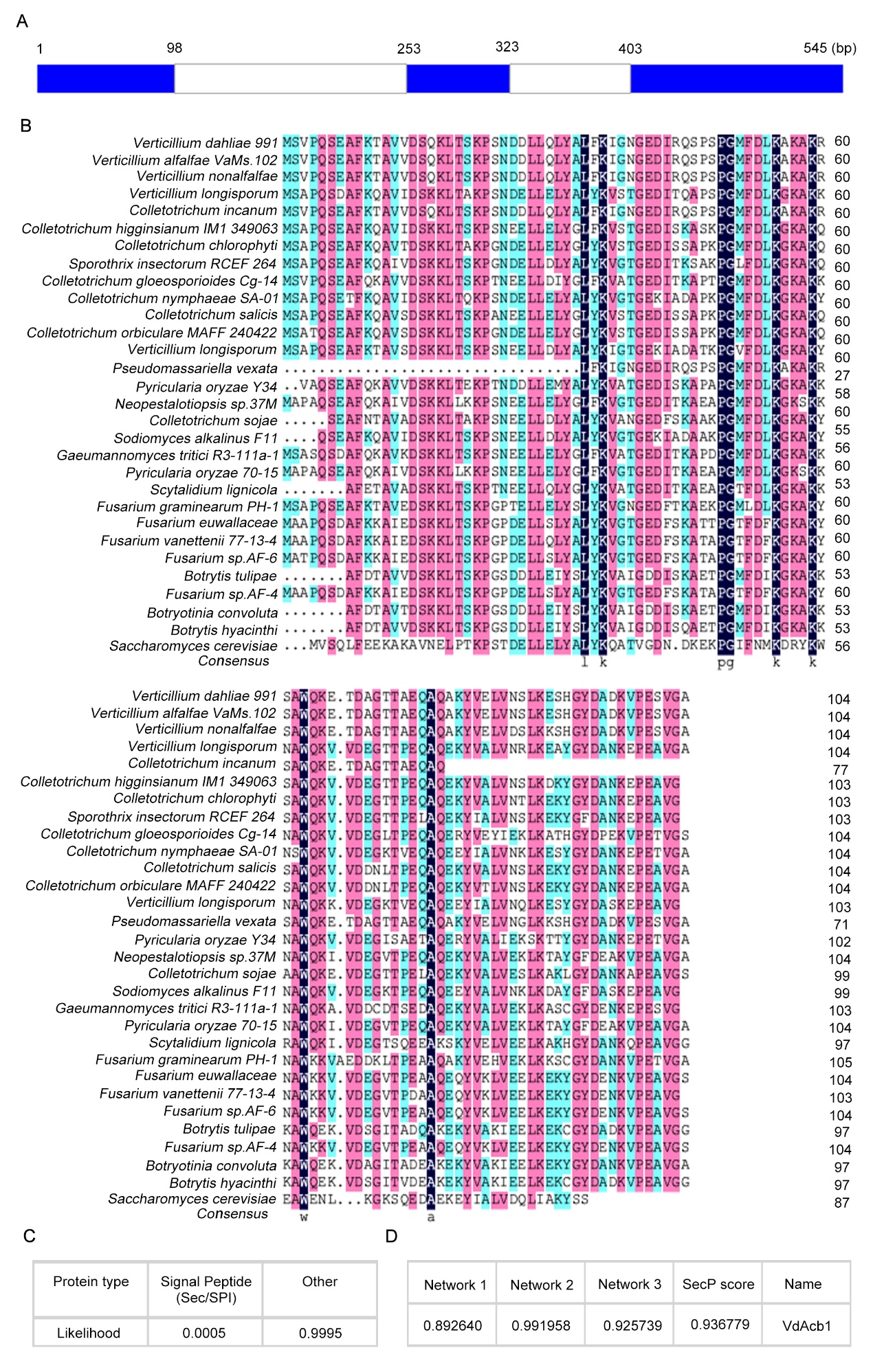
**

**Fig. S1** **Bioinformatic analysis of VdAcb1 in *V. dahliae*.** **A** Gene structure of *VdAcb1*. Blue boxes represent the exons and white boxes represent introns. **B** Sequence alignment of VdAcb1 homologues used to prepare the phylogenetic tree **C** Signal peptide prediction of VdAcb1 using SignalP5.0. **D** Unconventional secretion feature of VdAcb1 using SecretomeP 2.0. Network 1, score of amino acid composition; Network 2, score of secondary structure prediction; Network 3, score of transmembrane helix prediction; SepP score, the average of three network scores.

**
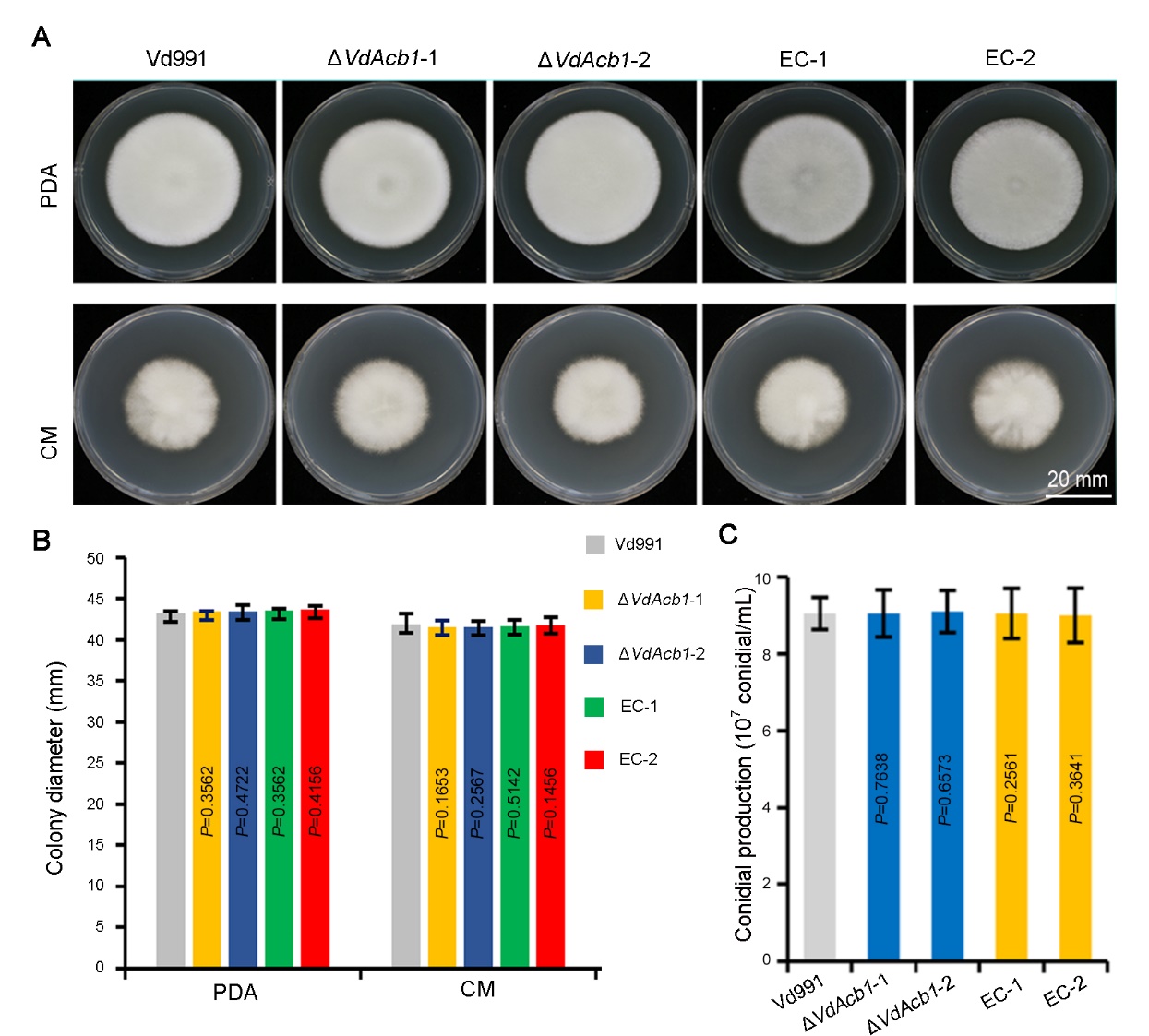
**

**Fig. S2** **Analyses of VdAcb1 in development and penetration A** Colony morphology of wild type (Vd991), Δ*VdAcb1*, and EC strains on PDA, CM and MM medium after culture at 25°C for 9 days. Scale bar = 20 mm. **B** Colony diameter of the different strains grown on different mediums for 9 days. Error bars represent the standard deviation of three biological replicates. Statistical significance was calculated by Student *t*-test. **C** The effect of VdAcb1 on conidial production of *V. dahliae*. After the strain was grown on PDA medium for 9 days, three agar plugs were made at the edge of the colony with a 6 mm-puncher and placed in 1 mL sterile water. Error bars represent the standard deviation between triplicate experiments. Statistical significance was calculated by Student *t*-test.

**
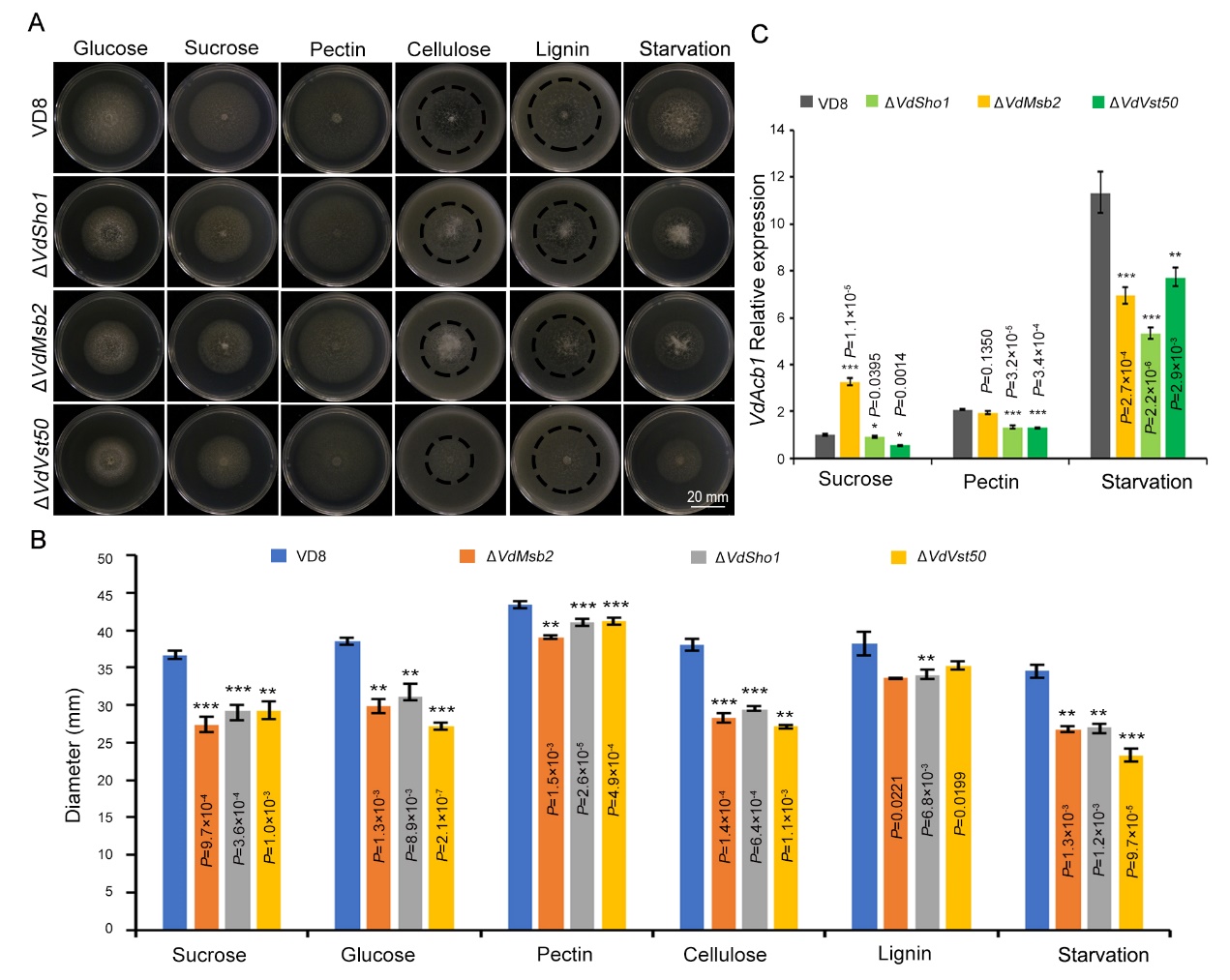
**

**Fig. S3 Growth phenotype and expression of *VdAcb1* analysis of wild-type (VD8), Δ*VdSho1,* Δ*VdMsb2* and Δ*VdVst50* in different carbon source medium. A** Colony morphology of wild-type (VD8), Δ*VdSho1,* Δ*VdMsb2* and Δ*VdVst50* grown on Czapek salt medium containing glucose, sucrose, cellulose, pectin, lignin and no carbon sources (stravation) at 25℃ for 9 days. Scale bar = 20 mm. **B** Colony diameter of indicated strains grown in different carbon source conditions for 9 days. Error bars represent the standard deviation of three biological replicates. Statistical significance was calculated by Student *t*-test with * and ** representing significance at *P* < 0.05 and *P* < 0.01, respectively. **C** RT-qPCR analysis of *VdAcb1* expression in wild-type (VD8), Δ*VdSho1*, Δ*VdMsb2* and Δ*VdVst50* strains that were cultured on Czapek salt medium supplemented with sucrose and pectin or Czapek salt medium only (starvation) for 4 days. The expression level of *VdAcb1* in the wild-type (VD8) was set to 1 and *VdEF-1α* was used as an endogenous control for gene expression analysis. Error bars represent the standard deviation between triplicate experiments. Statistical significance was calculated by an unpaired Student *t*-test with *, ** and *** representing significance at *P* < 0.05, *P* < 0.01 and *P* < 0.001, respectively.

**
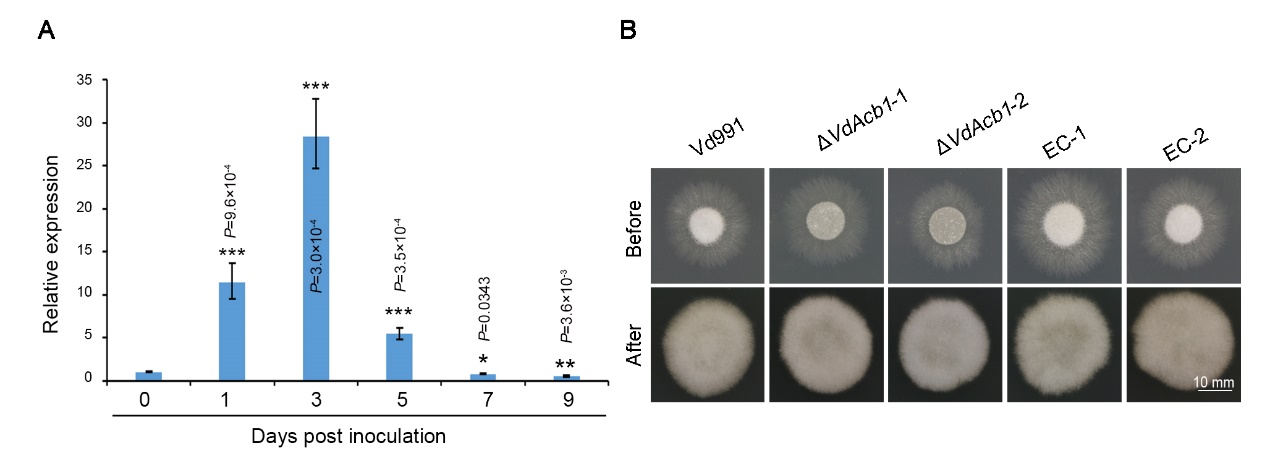
**

**Fig. S4** **Analysis of expression of *VdAcb1* during cotton root infection and analysis of penetration of the VdAcb1 deletion mutant. A** Expression levels were determined at the indicated time points by RT-qPCR and are given relative to *VdAcb1* expression values in conidia (time point 0). Error bars represent the standard deviation between triplicate experiments. Statistical significance was calculated by an unpaired Student *t*-test. *, ** and *** represent *P* < 0.05, *P* < 0.01 and *P* < 0.001 respectively. **B** Penetration analysis of the indicated *V. dahliae* strains. The Vd991, Δ*VdAcb1*, and EC were cultured on the MM medium covered with cellophane for 4 days at 25 ℃ (“Before” status). The culture was allowed to incubate and additional 4 days after removing cellophane from the medium and the growth of mycelium was observed (“After” status). Scale bar = 10 mm.

| **Table S1 Primers used in this study** | | |
| --- | --- | --- |
| **Primer name** | **Primer sequence (5′-3′)** | **Purpose** |
| ***VdAcb1* gene cloning** | |  |
| VdAcb1-DNA-F | ATGTCTGTTCCCCAGAGCGA | *VdAcb1* DNA sequences cloning |
| VdAcb1-DNA-R | GGCGCCGACGGACTCGGGAA | *VdAcb1* DNA sequences cloning |
| VdAcb1-F | ATGTCTGTTCCCCAGAGC | *VdAcb1* cDNA sequences cloning |
| VdAcb1-R | GGCGCCGACGGACTCGGG | *VdAcb1* cDNA sequences cloning |
| **Cloning *VdAcb1* signal peptide** | |  |
| SP-VdAcb1-F | GGAATTTTAATTAAGAATTCATGTCTGTTCCCCAGAGCGA | *VdAcb1* 40 aa N-terminal sequence clonging |
| SP-VdAcb1-R | CTATAGGGAGAACCTCGAGCTTCCAAGCCGAGCAACGAC | *VdAcb1* 40 aa N-terminal sequence clonging |
| ***VdAcb1* deletion** |  |  |
| VdAcb1-UP-F | AGGGTTGTGGTGCGTCGCATAG | To amplify upstream DNA fragment of *VdAcb1* |
| VdAcb1-UP-R | GCCCAAAAATGCTCCTTCAATCGGCTTGGAAGTGAGCTTCTGG | To amplify upstream DNA fragment of *VdAcb1* |
| VdAcb1-Down-F | CCCTGGGTTCGCAAAGATAAACAGCCTCAAGGAGTCTCACGG | To amplify downstream DNA fragment of *VdAcb1* |
| VdAcb1-Down-R | ACGGAGCAACTGGTCAAGTACGAT | To amplify downstream DNA fragment of *VdAcb1* |
| VdAcb1-Nest-F | GAGGTCGACGGTATCGATAAGCTTGGCAGTTGAACATGAAAGG | Nest PCR primer to obtain the amplicon |
| VdAcb1-Nest-R | TCCACCGCGGTGGCGGCCGCTCTAGATCAAGCGGGAAAGTCAGAGGT | Nest PCR primer to obtain the amplicon |
| VdAcb1-Test-F | CTGCCTCCAACTTTGTCCTCAAC | mutant detection, amplication of a part of *VdAcb1* sequence |
| VdAcb1-Test-R | GGGCGTTTCGTAATCGTCACTC | mutant detection, amplication of a part of *VdAcb1* sequence |
| VdAcb1-UP-F | AGGGTTGTGGTGCGTCGCATAG | To amplify upstream DNA fragment of *VdAcb1* |
| VdAcb1-UP-R | GCCCAAAAATGCTCCTTCAATCGGCTTGGAAGTGAGCTTCTGG | To amplify upstream DNA fragment of *VdAcb1* |
| ***VdMsn2* deletion** |  |  |
| VdMsn2-UP-F | CCGTCACCAGCCCCTGGGTTGAATTCTCGTATGGACTGGTGGGTTC | To amplify upstream DNA fragment of *VdMsn2* |
| VdMsn2-UP-R | ATGCTCCTTCAATATCAGAATTCTTTCGGTCCTTGACGTCTGA | To amplify upstream DNA fragment of *VdMsn2* |
| VdMsn2-Down-F | AGATGCCGACCGGGAACCAGAGCTCGACCCAGCTCTCTCTGCATT | To amplify downstream DNA fragment of *VdMsn2* |
| VdMsn2-Down-R | GCATGCCTGCAGGTCGACTCTAGAATGAGACATGTAATGTTCTATC | To amplify downstream DNA fragment of *VdMsn2* |
| VdMsn2-Test-F | CTTCCCTACGTCGTCCACTG | mutant detection, amplication of a part of *VdMsn2* sequence |
| VdMsn2-Test-R | ATGAAGCTAGCGTGACCGAG | mutant detection, amplication of a part of *VdMsn2* sequence |
| ***VdMsn4* deletion** |  |  |
| VdMsn4-UP-F | ATGGACGTAGAGCATGACGC | To amplify upstream DNA fragment of *VdMsn4* |
| VdMsn4-UP-R | GCCCAAAAATGCTCCTTCAAGACATACCTGCGGTCAAAGC | To amplify upstream DNA fragment of *VdMsn4* |
| VdMsn4-Down-F | CCCTGGGTTCGCAAAGATAAACGGTGTCGAGGAAGAGGTA | To amplify downstream DNA fragment of *VdMsn4* |
| VdMsn4-Down-R | TCAAGACAAAGGAGGGCGAC | To amplify downstream DNA fragment of *VdMsn4* |
| VdMsn4-Nest-F | GAGGTCGACGGTATCGATAAGCTTTTCATCTCGATCCACTCGGC | Nest PCR primer to obtain the amplicon |
| VdMsn4-Nest-R | CCAATTTGAGTACCCAATTCGAATTCGATACGCTGAGCAACTCCGA | Nest PCR primer to obtain the amplicon |
| VdMsn4-Test-F | CGCTGCACGCAGAAAAGATT | mutant detection, amplication of a part of *VdMsn4* sequence |
| VdMsn4-Test-R | ATACGTGGGGTGTTTCCTCG | mutant detection, amplication of a part of *VdMsn4* sequence |
| HYG-F | TTGAAGGAGCATTTTTGGGC | To amplify hygromycin resistent cassette DNA sequence |
| HYG-R | TTATCTTTGCGAACCCAGGG | To amplify hygromycin resistent cassette DNA sequence |
| ***VdAcb1* complementation** | |  |
| VdAcb1-C-F | AGCTCGGTACCCGGGGATCCTCTAGAACCTTCTTGGCGATGGAGTG | amplication of gene *VdAcb1* with flanking sequence |
| VdAcb1-C-R | TTGCATGCCTGCAGGTCGACTCTAGATCAAGCCTTCATCGTGCCAT | amplication of gene *VdAcb1* with flanking sequence |
| **VdAcb1-HA overexpression** | |  |
| VdAcb1-HA-F | TACCCAAGCATCGATGAGCTCATGTCTGTTCCCCAGAGC | To overexpress VdAcb1-HA for Western blotting |
| VdAcb1-HA-R | AACGTTAAGTGGATCTCTAGATTAGGCGTAGTCAGGCACGTCGTAAGGATAGGCGCCGA | To overexpress VdAcb1-HA for Western blotting |
| **VdAcb1-GFP overexpression** | |  |
| VdAcb1-GFP-F | ACCCAAGCATCGATGGTACCATGTCTGTTCCCCAGAGC | To overexpress VdAcb1-GFP for Confocal scanning |
| VdAcb1-GFP-R | CCCTTGCTCACCATGGTACCGGCGCCGACGGACTCGGG | To overexpress VdAcb1-GFP for Confocal scanning |
| Geneticin-F | ATGATTGAACAAGATGGATTG | Overexpressing strains detection, amplication of a part of geneticin resistent cassette DNA sequence |
| Geneticin-R | TCAGAAGAACTCGTCAAGAAGG | Overexpressing strains detection, amplication of a part of geneticin resistent cassette DNA sequence |
| **Fungal biomass detection** | |  |
| EF-1α-F | TGAGTTCGAGGCTGGTATCT | *V.dahliae EF-1α* gene used for fungal biomass detection |
| EF-1α-R | CACTTGGTGGTGTCCATCTT | *V.dahliae EF-1α* gene used for fungal biomass detection |
| 18S-F | CGGCTACCACATCCAAGGAA | Cotton *18S* gene used for fungal biomass detection |
| 18S-R | TGTCACTACCTCCCCGTGTCA | Cotton *18S* gene used for fungal biomass detection |
| NbEF1-α-F | TGAGTTCGAGGCTGGTATCT | *Nicotiana benthamiana EF-1α* gene used for fungal biomass detection |
| NbEF1-α-R | CACTTGGTGGTGTCCATCTT | *Nicotiana benthamiana EF-1α* gene used for fungal biomass detection |
| AtUBQ1-F | TTCCTTGATGATGCTTGCTC | *Arabidopsis thaliana UBQ1* gene used for fungal biomass detection |
| AtUBQ1-R | TTGACAGCTCTTGGGTGAAG | *Arabidopsis thaliana UBQ1* gene used for fungal biomass detection |
| **Yeast one-hybrid** |  |  |
| VdMsn2-AD-F | GCCATGGAGGCCAGTGAATTCATGGACCCCAACATGATGAC | To construct the VdMsn2-pGADT7 vector |
| VdMsn2-AD-R | ATGCCCACCCGGGTGGAATTCTTACTCAGCTCGCTTGCGCT | To construct the VdMsn2-pGADT7 vector |
| VdMsn4-AD-F | GCCATGGAGGCCAGTGAATTCATGGCTTCGAACGGTGCCCC | To construct the VdMsn4-pGADT7 vector |
| VdMsn4-AD-R | ATGCCCACCCGGGTGGAATTCCTACCTCTTCCTCGACACCG | To construct the VdMsn4-pGADT7 vector |
| **qPCR** |  |  |
| qAcb1-F | TCTTCAAGATTGGCAACGGC | Primer used for qPCR measurement of *VdAcb1* gene transcription level in *V. dahliae* |
| qAcb1-R | GGACTCGGGAACCTTATCGG | Primer used for qPCR measurement of *VdAcb1* gene transcription level in *V. dahliae* |
| qMsn2-F | CAGTCTTTGACCGAGGACCC | Primer used for qPCR measurement of *VdMsn2* gene transcription level in *V. dahliae* |
| qMsn2-R | TGAGATCCATGACGATGGCG | Primer used for qPCR measurement of *VdMsn2* gene transcription level in *V. dahliae* |
| qMsn4-F | GCCATCATCAAGTTCCGCAC | Primer used for qPCR measurement of *VdMsn4* gene transcription level in *V. dahliae* |
| qMsn4-R | CGGGCTTCTCCTTAGGAACC | Primer used for qPCR measurement of *VdMsn4* gene transcription level in *V. dahliae* |
| VEDA_04969-F | CGTCCCTTCTTTGGTCTTCTC | Primer used for qPCR measurement of *VEDA_04969* gene transcription level in *V. dahliae* |
| VEDA_04969-R | TAGAGGTAGAAGGAGACGTTGG | Primer used for qPCR measurement of *VEDA_04969* gene transcription level in *V. dahliae* |
| VEDA_01382-F | CCGACGAACACCAAGATGTA | Primer used for qPCR measurement of *VEDA_01382* gene transcription level in *V. dahliae* |
| VEDA_01382-R | CAGTTGGAGCCTTGGTAGTAG | Primer used for qPCR measurement of *VEDA_01382* gene transcription level in *V. dahliae* |
| VEDA_09412-F | GAGGGCAGTGTTGACTTTATTTG | Primer used for qPCR measurement of *VEDA_09412* gene transcription level in *V. dahliae* |
| VEDA_09412-R | GTCTGTTCTGGGCAGTAATGTA | Primer used for qPCR measurement of *VEDA_09412* gene transcription level in *V. dahliae* |
| VEDA_05701-F | CTTCATCAACCAGGGCTCATAC | Primer used for qPCR measurement of *VEDA_05701* gene transcription level in *V. dahliae* |
| VEDA_05701-R | TCGCCGTAATGGTGACTTTG | Primer used for qPCR measurement of *VEDA_05701* gene transcription level in *V. dahliae* |
| VEDA_07932-F | ATGAAGTTCCTCAGCCTCATC | Primer used for qPCR measurement of *VEDA_07932* gene transcription level in *V. dahliae* |
| VEDA_07932-R | TCACCAGTGAGGACAAAGTAAG | Primer used for qPCR measurement of *VEDA_07932* gene transcription level in *V. dahliae* |
| VEDA_05702-F | AAGGGCGACTACGAAGATATTG | Primer used for qPCR measurement of *VEDA_05702* gene transcription level in *V. dahliae* |
| VEDA_05702-R | CCAGTGTCGTGTAAGTGTTTAG | Primer used for qPCR measurement of *VEDA_05702* gene transcription level in *V. dahliae* |
| VEDA_03250-F | CCAACAGCACAAACGTCATC | Primer used for qPCR measurement of *VEDA_03250* gene transcription level in *V. dahliae* |
| VEDA_03250-R | CGACCACGTCGAAGAACTC | Primer used for qPCR measurement of *VEDA_03250* gene transcription level in *V. dahliae* |
| VEDA_01417-F | CTGGATGCTCAACGGTAACA | Primer used for qPCR measurement of *VEDA_01417* gene transcription level in *V. dahliae* |
| VEDA_01417-R | CGTCGTTGGCCGAATAGAA | Primer used for qPCR measurement of *VEDA_01417* gene transcription level in *V. dahliae* |
| VEDA_01470-F | GGATGGCTACACGATCCTAAAC | Primer used for qPCR measurement of *VEDA_01470* gene transcription level in *V. dahliae* |
| VEDA_01470-R | CCCAGTAGTTGTATGCCTCATT | Primer used for qPCR measurement of *VEDA_01470* gene transcription level in *V. dahliae* |
| VEDA_03111-F | CGAGGGCGAGAACATCTTT | Primer used for qPCR measurement of *VEDA_03111* gene transcription level in *V. dahliae* |
| VEDA_03111-R | CAGACCCTTGGTGCCATATT | Primer used for qPCR measurement of *VEDA_03111* gene transcription level in *V. dahliae* |
| VEDA_07591-F | AACGAAACCTCAGACTCCTTC | Primer used for qPCR measurement of *VEDA_07591* gene transcription level in *V. dahliae* |
| VEDA_07591-R | GTTGGACGCTGAGAAGTAGAA | Primer used for qPCR measurement of *VEDA_07591* gene transcription level in *V. dahliae* |
| VEDA_07914-F | ACACCTTTACCCTCACCTTTG | Primer used for qPCR measurement of *VEDA_07914* gene transcription level in *V. dahliae* |
| VEDA_07914-R | ACCGTTCTTGCGGTTCTT | Primer used for qPCR measurement of *VEDA_07914* gene transcription level in *V. dahliae* |
| VEDA_07694-F | GCTGTCTACTACAACCTCACAG | Primer used for qPCR measurement of *VEDA_07694* gene transcription level in *V. dahliae* |
| VEDA_07694-R | GATGAACGACGAGATGGTATCC | Primer used for qPCR measurement of *VEDA_07694* gene transcription level in *V. dahliae* |
| VEDA_01287-F | GCCCAACATGCTCCTCTAC | Primer used for qPCR measurement of *VEDA_01287* gene transcription level in *V. dahliae* |
| VEDA_01287-R | CTCGGTGAGGTTGATCGTATTT | Primer used for qPCR measurement of *VEDA_01287* gene transcription level in *V. dahliae* |
| VEDA_02509-F | CGAGTTCACCTCCGACATTAC | Primer used for qPCR measurement of *VEDA_02509* gene transcription level in *V. dahliae* |
| VEDA_02509-R | CTTCCAGTGGTCGTGGAAATAG | Primer used for qPCR measurement of *VEDA_02509* gene transcription level in *V. dahliae* |
| VEDA_05049-F | GGCAAGCTCCACGTTACTTA | Primer used for qPCR measurement of *VEDA_05049* gene transcription level in *V. dahliae* |
| VEDA_05049-R | CAATGAGACCGTTCCAGTAGTT | Primer used for qPCR measurement of *VEDA_05049* gene transcription level in *V. dahliae* |
| VEDA_08848-F | CGCTTTACAACACCACAACAG | Primer used for qPCR measurement of *VEDA_08848* gene transcription level in *V. dahliae* |
| VEDA_08848-R | ATAGTCAGCAAGCCACTCATAC | Primer used for qPCR measurement of *VEDA_08848* gene transcription level in *V. dahliae* |
| VEDA_05899-F | GACGCCATTTCCATCAAGAAC | Primer used for qPCR measurement of *VEDA_05899* gene transcription level in *V. dahliae* |
| VEDA_05899-R | TATGCTGCACGACCTTATCC | Primer used for qPCR measurement of *VEDA_05899* gene transcription level in *V. dahliae* |
| VEDA_07870-F | ATACCCTCGTCTTCTCCGTATC | Primer used for qPCR measurement of *VEDA_07870* gene transcription level in *V. dahliae* |
| VEDA_07870-R | GGGTGCCTTGAGAAGCATATT | Primer used for qPCR measurement of *VEDA_07870* gene transcription level in *V. dahliae* |
| qVst7-F | GTGCTGCCACCTTTAGCCTCCC | Primer used for qPCR measurement of *Vst7* gene transcription level in *V. dahliae* |
| qVst7-R | CGTCATTCATGTAGCCTCTGTTGGA | Primer used for qPCR measurement of *Vst7* gene transcription level in *V. dahliae* |
| qVst11-F | GTCAGACGAGGCACTGGAGG | Primer used for qPCR measurement of *Vst11* gene transcription level in *V. dahliae* |
| qVst11-R | TGTCGGACAAAGCTGCGCAC | Primer used for qPCR measurement of *Vst11* gene transcription level in *V. dahliae* |
| qVst20-F | GCACTCAAAGGGCGTTATCC | Primer used for qPCR measurement of *Vst20* gene transcription level in *V. dahliae* |
| qVst20-R | CCAGTAAGGAGTGCCGACCAT | Primer used for qPCR measurement of *Vst20* gene transcription level in *V. dahliae* |
| qVst50-F | ACGCAACACGAACGCTCCAT | Primer used for qPCR measurement of *Vst50* gene transcription level in *V. dahliae* |
| qVst50-R | GGTGCGGGATGAAGGTGTCG | Primer used for qPCR measurement of *Vst50* gene transcription level in *V. dahliae* |
| qCdc42-F | CGTATGCTTCAGCGTCACCT | Primer used for qPCR measurement of *Cdc42* gene transcription level in *V. dahliae* |
| qCdc42-R | CGTACTTGACCGCACCGAGA | Primer used for qPCR measurement of *Cdc42*  gene transcription level in *V. dahliae* |
| qELF-F | CCATTGATATCGCACTGTGG | Primer used for qPCR measurement of *EF-1α* gene transcription level in *V. dahliae* |
| qELF-R | TGGAGATACCAGCCTCGAAC | Primer used for qPCR measurement of *EF-1α* gene transcription level in *V. dahliae* |
| qSho1-F | CTATGTCCTGCTGTCGGTTGTC | Primer used for qPCR measurement of *Sho1* gene transcription level in *V. dahliae* |
| qSho1-R | TGGAGGCTGGACCGAAGTGG | Primer used for qPCR measurement of *Sho1* gene transcription level in *V. dahliae* |
| qMsb2-F | CTGGCGATACGGAGAATGTTG | Primer used for qPCR measurement of *Msb2* gene transcription level in *V. dahliae* |
| qMsb2-R | ATGGGCAACAGCGTGAACAA | Primer used for qPCR measurement of *Msb2* gene transcription level in *V. dahliae* |

| **Table S2 The gene ID of sugar transport-related proteins in Vd991** | |
| --- | --- |
| **Gene ID** | **Gene name** |
| VEDA_03709 | MFS hexose transporter |
| VEDA_05498 | H+/hexose cotransporter 1 |
| VEDA_08768 | quinate permease |
| VEDA_07223 | sugar transporter |
| VEDA_02598 | quinate permease |
| VEDA_09757 | alpha-glucosides permease MPH2/3 |
| VEDA_05908 | quinate permease |
| VEDA_02565 | high-affinity glucose transporter RGT2 |
| VEDA_07558 | quinate permease |
| VEDA_06849 | maltose permease MAL31 |
| VEDA_01421 | sugar transporter STL1 |
| VEDA_03714 | alpha-glucosides permease MPH2/3 |
| VEDA_04927 | H+/hexose cotransporter 1 |
| VEDA_01012 | galactose-proton symporter |
| VEDA_00014 | maltose permease |
| VEDA_06986 | sugar transporter |
| VEDA_07927 | maltose permease MAL31 |
| VEDA_07879 | high-affinity glucose transporter RGT2 |
| VEDA_05987 | sugar transporter STL1 |
| VEDA_06526 | sugar transporter |
| VEDA_07182 | alpha-glucosides permease MPH2/3 |
| VEDA_08198 | sugar transporter |
| VEDA_03844 | galactose transporter |
